# Divergent acetyl-CoA binding modes mediate allosteric inhibition of bacterial hybrid-type malic enzymes

**DOI:** 10.1016/j.jbc.2025.110887

**Published:** 2025-11-04

**Authors:** Munetoshi Sassa, Haruka Yamato, Hiroki Tanino, Yohta Fukuda, Tsuyoshi Inoue

**Affiliations:** 1 Graduate School of Pharmaceutical Sciences, The University of Osaka, Suita, Japan; 2 Integrated Frontier Research for Medical Science Division, Institute for Open and Transdisciplinary Research Initiatives (OTRI), The University of Osaka, Suita, Japan; 3 Center for Infectious Disease Education and Research (CiDER), The University of Osaka, Suita, Japan

**Keywords:** oxidoreductases, malic enzyme, X-ray crystallography, cryo-electron microscopy, allostery

## Abstract

Malic enzymes (MEs) function as the bypass enzyme in the Krebs cycle and have attracted attention in a wide range of scientific and industrial fields. In contrast to eukaryotic MEs, there is currently a lack of understanding of the structure-function relationships of prokaryotic MEs. Especially, little is known about an allosteric inhibition mechanism by an effector ligand in multi-domain MEs called hybrid-type MEs. Many bacterial hybrid-type MEs are inhibited by acetyl-CoA; however, the proposed acetyl-CoA binding site is not conserved. Here, we determined crystal and cryo-EM structures of hybrid-type MEs from *Escherichia coli* (*Ec*MaeB) and *Bdellovibrio bacteriovorus* including complexes with acetyl-CoA. They reveal that these MaeBs have totally different acetyl-CoA binding sites and show different overall structural changes. However, the binding acetyl-CoA molecules induce identical movements of several α helices near the ligand both in *Ec*MaeB and *Bb*MaeB. Enzymatic assays proved that residues at the acetyl-CoA binding site are needed for inhibition. Phylogenetic analysis uncovered that *Ec*MaeB and *Bb*MaeB are classified into different clades of hybrid-type MEs and that the amino acid residues at the acetyl-CoA binding sites in different clades have evolved exclusively from each other. These results not only provide insights into bacterial MEs but also expand our knowledge about allosteric regulation in enzymes.

The tricarboxylic acid (TCA) cycle, commonly referred to as the Krebs cycle, serves as a crucial center of cellular metabolism, encompassing both catabolic and anabolic processes. In the TCA cycle, a series of chemical reactions that utilize high-energy electrons as an energy source are periodically repeated (1, 2, 3). As a bypass enzyme of the TCA cycle, malic enzymes (MEs) regulate cellular energy, redox balance, and biomolecular synthesis. While reducing NAD(P)^+^ to NAD(P)H, MEs catalyze the oxidative decarboxylation of the TCA cycle intermediate L-malate to produce the TCA carbon source pyruvate and a byproduct carbon dioxide (CO_2_) in the presence of divalent metal ions such as a magnesium (Mg^2+^) ion (4) (Fig. 1*A*). The reverse CO_2_ fixation reaction is also catalyzed by MEs. MEs are widespread in almost all organisms from eukaryotes to bacteria and archaea because the substrates and products of MEs are involved in various essential metabolic pathways. In fact, the chemical reaction of MEs is coupled with fundamental physiological processes: production of NADPH for biosynthesis of fatty acids (5, 6) and NADPH balancing (7), production of pyruvate for anabolic functions including photosynthesis and stress resistance (8, 9), and regulation of carbon flux (10, 11). Moreover, MEs regulate metabolism of glutamine as an alternative energy source in rapidly proliferating cells such as tumors (12, 13, 14). Human MEs are deeply involved in cancer and inflammatory diseases (15, 16).

**Figure 1:**
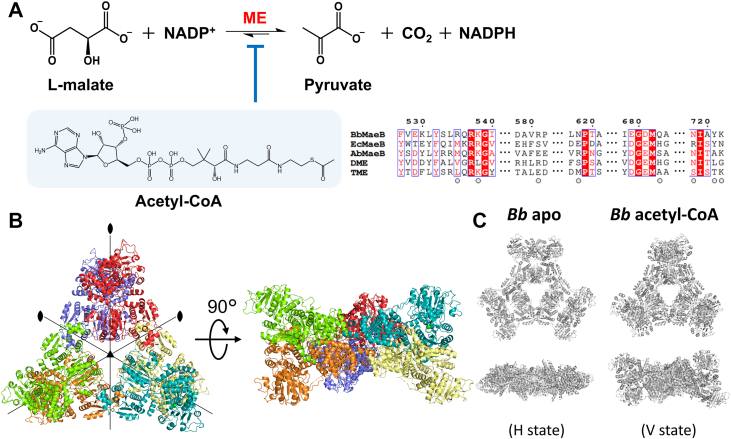
**Function and structure of *Ec*MaeB.***A*, schematic diagram for the reaction which malic enzymes (MEs) catalyze. An effector molecule acetyl-CoA decreases the enzymatic activity of hybrid-type MEs. Sequence alignments of hybrid-type MEs for representative bacteria studied to date. The open circles represent amino acids that were involved in acetyl-CoA binding in *Bb*MaeB. *B*, crystal structure of an *Ec*MaeB_apo_ hexamer. Each protomer is colored differently and shown by cartoon representation. The 2- and 3-fold axes are indicated by symmetry symbols. *C*, previously reported crystal structures of *Bb*MaeB apo form (PDB code ID: 6ZNJ) and *Bb*MaeB acetytl-CoA bound form (PDB code ID: 6ZNG). ME, malic enzyme.

On top of such basic biological importance, MEs are also attractive enzymes in industrial fields. For example, attempts are being made to address global warming by developing CO_2_ fixation technology using MEs (17, 18). Optimal enzymatic activity of MEs is essential for ethanol production by *Synechocystis* sp. PCC 6803, a photosynthetic microorganism that produces useful chemicals from CO_2_ and sunlight (19). In the field of food chemistry, the influence of MEs on the protein and lipid composition on crops has been studied. Although it is generally difficult to improve the composition of soybeans (20, 21, 22, 23, 24, 25, 26), it could be altered by increasing the activity of MEs (27). In the field of protein engineering, much work has been done to change the cofactor specificities of MEs (28, 29) and to improve stability of the enzyme (30).

With these basic and applied scientific motivations as a background, structural biology to understand catalytic mechanism of MEs has been vigorously conducted. Especially, structures of MEs from eukaryotes, isoforms of which are found in cytoplasm (31, 32, 33), mitochondria (34, 35), and pigment bodies (36), are extensively studied (31, 33, 37, 38, 39, 40, 41). In contrast, structures of prokaryotic MEs are comparatively unexplored. This is partly because MEs from prokaryotes are more structurally diverse. Molecular mass of prokaryotic MEs ranges from 40 kDa to more than 85 kDa having multi-domains (42, 43, 44, 45, 46, 47, 48). Because many industrial applications are focusing on prokaryotic MEs, understanding of their structure-function relationships is coveted. Studying bacterial MEs also has potential for the development of new antimicrobial agents. It is recently showed that an ME from *Mycobacterium tuberculosis*, which is involved in maintaining the integrity of the cell wall, can be a drug target (49).

The most complicated type of prokaryotic MEs is hybrid-type MEs, which is a fusion of unrelated domains. A diphosphopyridine nucleotide-dependent malic enzyme (DME) and a triphosphopyridine nucleotide-dependent malic enzyme (TME) from *Ensifer meliloti* (formerly *Sinorhizobium meliloti*) (50) are the firstly discovered hybrid-type MEs. The genome of a model bacterium *Escherichia coli* codes 2 ME genes and one of them is a hybrid-type ME called malic enzyme B (MaeB) (51). The hybrid-type MEs consist of an N-terminal catalytic domain (ME domain) and a C-terminal activity regulating domain (50, 52). The latter is called phosphotransacetylase (PTA) domain because its amino acid sequence shows similarity to those of PTA family proteins. The PTA domain does not possess an enzymatic activity of PTA but acts as an allosteric modulator of catalysis; that is, binding of some metabolites to the PTA domain increases or decreases the enzymatic activity. The most studied effector molecule for hybrid-type MEs is acetyl coenzyme A (acetyl-CoA) (51, 53, 54). Binding of acetyl-CoA to the PTA domain is known to reduce the catalytic activity of several hybrid-type MEs.

Recently, crystal structures of a hybrid-type ME from a predatory bacterium *Bdellovibrio bacteriovorus* (*Bb*MaeB) have been reported. *Bb*MaeB assumes a homohexameric structure and its PTA domain contributes to oligomer formation and binding of acetyl-CoA (53). The study indicates that the binding of acetyl-CoA to the PTA domain of *Bb*MaeB induces structural changes with significant domain reorganization, resulting in inhibition of the chemical reaction. However, the amino acid sequence of the acetyl-CoA binding site in *Bb*MaeB is not conserved among those of other known hybrid-type MEs (Fig. 1*A*). Therefore, structural information on hybrid-type MEs from other bacteria provides more precise insights into their nature of the allosteric regulation. Here, we have performed structure determination of MaeB from *E. coli* (*Ec*MaeB) using X-ray crystallography and cryo-EM as well as enzymatic assays and phylogenetic analysis. Moreover, we determined cryo-EM structures of *Bb*MaeB with and without acetyl-CoA. Our comparative study shows that the same effector molecule binds to completely different sites in hybrid-type MEs from different organisms but causes the same local structural changes resulting in the same allosteric effect.

## Results

### Inhibition of *Ec*MaeB and *Bb*MaeB by acetyl-CoA

First, we conducted enzymatic assays for both *Ec*MaeB and *Bb*MaeB (Fig. S1*A*). While an earlier study employed *Bb*MaeB from *B*. *bacteriovorus* strain HD100 (*Bb*MaeB-HD), our analysis used the homolog from strain SSB218315 (*Bb*MaeB-SSB), which shares 97.6% amino acid identity with *Bb*MaeB-HD. The measured *K*_m_ values for NADP^+^ of *Ec*MaeB (24.9 ± 1.94 μM) and *Bb*MaeB (18.0 ± 1.52 μM) were consistent with previously reported data (51, 53), although the *k*_cat_ values for both enzymes were somewhat lower. A previous study reported that *Ec*MaeB activity is inhibited by Mg^2+^ at concentrations above 4 mM (51), but we observed no significant changes in kinetic parameters across different Mg^2+^ concentrations (Fig. S1*B*). When using an alternative divalent metal (Mn^2+^) ion, the *K*_m_ values for NADP^+^ of *Ec*MaeB and *Bb*MaeB approximately doubled while their *k*_cat_ values were unchanged (Fig. S1*C*). We employed 5 mM Mg^2+^ in our subsequent enzymatic assays.

High concentrations of the substrate malate were found to suppress MaeB activity (Fig. S1*D*) as reported previously (51, 53). We obtained *K*_m_ values for malate of *Ec*MaeB (20.7 ± 8.83 mM) and *Bb*MaeB (6.22 ± 1.55 mM) from the substrate inhibition plots, which were consistent with previously reported data (51, 53). To avoid substrate inhibition, we performed acetyl-CoA inhibition assays described below under low-malate conditions.

As expected, both *Ec*MaeB and *Bb*MaeB exhibited sensitivity to acetyl-CoA (Fig. S1*E*). The IC_50_ for *Bb*MaeB-SSB (0.131 ± 0.004 μM) closely matched that of *Bb*MaeB-HD (0.453 ± 0.019 μM) and was markedly lower than that of *Ec*MaeB (102 ± 4.92 μM), indicating that *Bb*MaeB is substantially more susceptible to inhibition by acetyl-CoA. Hill slope factors derived from the IC_50_ curves were greater than one for both enzymes, implying cooperative behavior and the involvement of an allosteric regulatory mechanism. In the presence of acetyl-CoA, *Bb*MaeB exhibited significantly reduced reaction rates and displayed a sigmoidal kinetic curve (Fig. S1*F*). The *K*_0.5_ value obtained from the empirical Hill equation (7.44 ± 1.21 mM) was comparable to the *K*_m_ value observed in the absence of acetyl-CoA, indicating that the inhibition by the effector is non-competitive. Similarly, acetyl-CoA decreased the reaction rate of *Ec*MaeB in the low-malate concentration range, while its *K*_0.5_ value (18.1 ± 3.20 mM) was essentially unchanged from the *K*_m_ value. Although this result suggests that *Ec*MaeB inhibition by acetyl-CoA may follow a non-competitive mode, we cannot conclude that *Ec*MaeB is inhibited exclusively through this mechanism, given the difficulty in determining a reliable *V*_max_ under the present experimental conditions. At higher malate concentrations, the reaction rates of *Ec*MaeB approached those observed without acetyl-CoA, indicating that *Ec*MaeB becomes less responsive to the effector under conditions of substrate saturation. Because substrate inhibition also occurs at the same malate-concentration region, it was difficult to accurately evaluate the inhibitory effect of acetyl-CoA on *Ec*MaeB activity and to determine reliable kinetic parameters under these conditions.

The marked difference in acetyl-CoA sensitivity between *Ec*MaeB and *Bb*MaeB implies that the two enzymes recognize acetyl-CoA differently, likely due to variations in their binding sites and/or binding mechanisms. Since enzymatic measurements alone cannot fully resolve the precise inhibition mode, structural analyses were required to clarify the molecular mechanisms underlying *Ec*MaeB and *Bb*MaeB regulation.

### Structural analysis of *Ec*MaeB without acetyl-CoA

For structural analysis of *Ec*MaeB, we initially determined the X-ray crystal structure of *Ec*MaeB without cofactors (NADP^+^ and Mg^2+^) and the effector ligand acetyl-CoA (*Ec*MaeB_apo_) at 3.85 Å resolution (Fig. 1*B*). There are two almost identical homohexamer of *Ec*MaeB in the asymmetric unit although *Ec*MaeB was previously expected to form a homooctamer based on the results of size exclusion chromatography and native electrophoresis (51). The homohexameric state is also observed in *Bb*MaeB (53). The resolution of our crystallographic data is not so high, but electron density of all subdomains was clearly observed (Fig. S2). The subdomain architecture of *Ec*MaeB (crossover, d1, d2, and hook subdomains in the ME domain and d1, d2, and 3′ loop subdomains in the PTA domain) is identical to *Bb*MaeB (Fig. S3). The *Ec*MaeB_apo_ homohexamer placed the PTA domain and the ME domain on the inside and the outside of the hexameric structure, respectively, as the *Bb*MaeB homohexamer does. The hexamer shows the *D*3 symmetry, where the 3-fold axis is located at the center of a triangle formed by the PTA domains and 2-fold axes at around the interfaces of four protomers. Here, we define the state represented by this homohexamer as a vertical (V) state because the ME domain dimers are almost vertical to the plane on which 2-fold axes exist. Contrary to our expectations, superposition of the *Ec*MaeB_apo_ structure on crystal structures of *Bb*MaeB reveals that the *Ec*MaeB_apo_ structure is more similar to the acetyl-CoA bound form of *Bb*MaeB showing a V state (PDB code ID: 6ZNG) than the *Bb*MaeB apo form assuming a horizontal (H) state in which the ME domain dimers are horizontally placed on the plane on which 2-fold axes exist (PDB code ID: 6ZNJ) (Fig. 1*C*).

**Figure 2:**
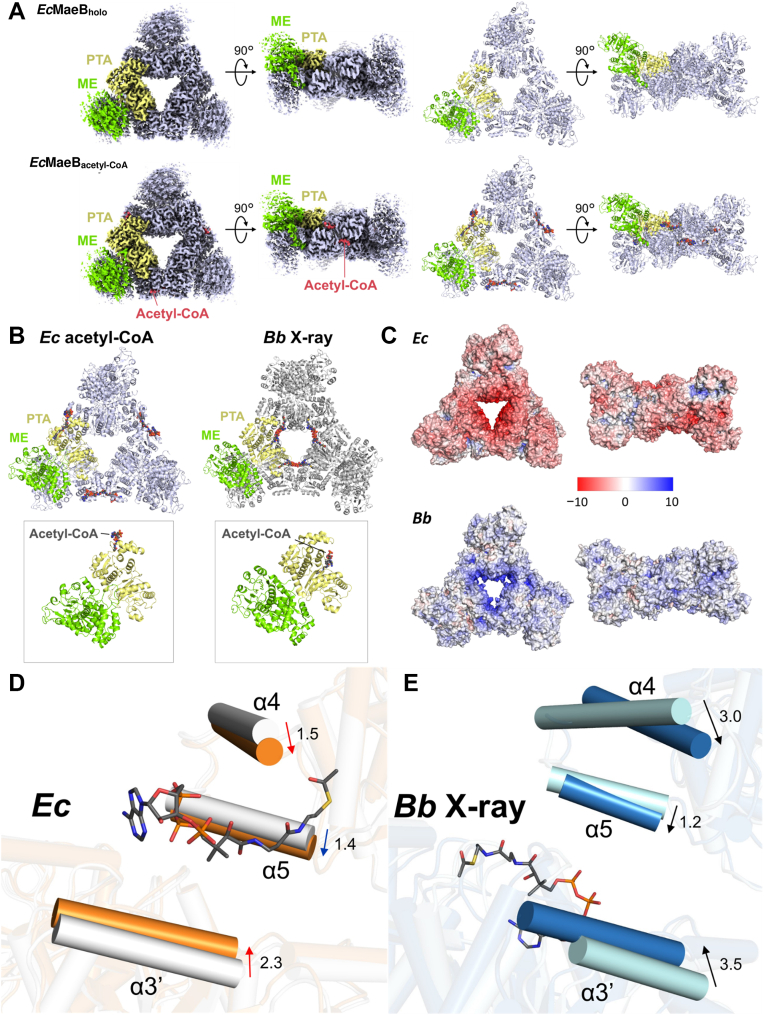
**Cryo-EM structures of *Ec*MaeB.***A*, the cryo-EM map (*left*) and structural model (*right*) of *Ec*MaeB_holo_ (*upper*) and *Ec*MaeB_acetyl-CoA_ (*lower*). The ME domain and the PTA domain are colored *green* and *yellow*, respectively. Acetyl-CoA is colored *red* in the cryo-EM map (*left*) and is shown by spheres in the model (*right*). *B*, structural comparison of the binding site of acetyl-CoA between *Ec*MaeB_acetyl-CoA_ and *Bb*MaeB acetyl-CoA bound form (PDB code ID: 6ZNG). Acetyl-CoA is shown as sphere models colored by elements. *C*, electrostatic potential surfaces of *Ec*MaeB and *Bb*MaeB. The positively charged regions are colored *blue* and negatively are *red*. *D*, structural comparison of the acetyl-CoA binding site between *Ec*MaeB_holo_ (*white*) and *Ec*MaeB_acetyl-CoA_ (*orange*). For structural comparison, PTA domains in two protomers are superimposed (Cα RMSD = 1.09 Å over 654 atoms). *Red arrows* represent the change of helices approaching acetyl-CoA, and a *blue* arrow the movement of a helix away from acetyl-CoA. The numbers next to the arrows indicate the distance helices moved (Å). The shift distances for α3, α4, and α5 helices were determined by the migration distances of the Cα atoms of N477, E509, and R530, respectively. *E*, structural comparison of the acetyl-CoA binding site between *Bb*MaeB apo form (PDB code ID: 6ZNJ) (pale cyan) and *Bb*MaeB acetyl-CoA bound form (PDB code ID: 6ZNG) (*blue*). For structural comparison, PTA domains in two protomers are superimposed (Cα RMSD = 1.76 Å over 635 atoms). The numbers next to the arrows indicate the distance helices moved (Å). The shift distances for α3, α4, and α5 helices were determined by the migration distances of the Cα atoms of P495, F525, and R547, respectively. ME, malic enzyme.

The result that *Ec*MaeB_apo_ is the V state instead of the H state could be due to the effect of molecular packing in a crystal, which can limit possible protein structures. Therefore, we next performed cryo-EM analysis that can observe structures of protein particles dispersed in solution. To obtain a more physiologically relevant state structure, we added the cofactors NADP^+^ and MgCl_2_ to *Ec*MaeB_apo_ solution prior to data collection and determined its structure (*Ec*MaeB_holo_) at 2.73 Å resolution (Fig. 2*A*). The structure of *Ec*MaeB_holo_ is also the V state despite the absence of acetyl-CoA, like the crystal structure of *Ec*MaeB_apo_.

While the cryo-EM map of *Ec*MaeB_holo_ clearly shows the PTA domain, the map around the ME domain is unclear. Furthermore, in 2D classification of *Ec*MaeB_holo_ data, the ME domain was ambiguous (Fig. S4). These results of the cryo-EM map and 2D classification indicate the flexibility of the ME domain of *Ec*MaeB. Consequently, the catalytic site of *Ec*MaeB_holo_ including cofactors could not be modeled well although we added NADP^+^ and Mg^2+^ to the sample. Therefore, we had to conduct further local refinement of the ME domain dimer to observe cryo-EM maps of NADP^+^ and the Mg ion (Fig. S5). We calculated the RMSDs of Cα atoms between *Ec*MaeB_apo_ and *Ec*MaeB_holo_. For this structural comparison, the structure of PTA domain is used due to the ambiguity of the ME domain in *Ec*MaeB_holo_. The RMSD calculated using 1900 Cα atoms is 0.51 Å, showing that the PTA domains of *Ec*MaeB_holo_ and *Ec*MaeB_apo_ are almost identical to each other. Therefore, we use the higher resolution *Ec*MaeB_holo_ cryo-EM structure with bound cofactors for detailed structural comparison and discussion described below.

### Structural analysis of *Ec*MaeB acetyl-CoA bound form (*Ec*MaeB_acetyl-CoA_)

To find the acetyl-CoA binding site of *Ec*MaeB, we collected cryo-EM data of *Ec*MaeB_holo_ in the presence of acetyl-CoA (*Ec*MaeB_acetyl-CoA_) and determined its structure at 2.03 Å resolution (Fig. 2*A*). This structure reveals that *Ec*MaeB_acetyl-CoA_ forms the V state as does *Ec*MaeB_holo_. The cryo-EM map of the ME domain was ambiguous as is in the cryo-EM data of *Ec*MaeB_holo_ despite the higher resolution. As with *Ec*MaeB_holo_, local refinement of the ME domain dimer sharpened the NADP^+^ and Mg ion maps (Fig. S5, *B*–*D*). Acetyl-CoA molecules were located outside the apex of a triangle formed by the PTA domains. It is noteworthy that this binding site is different from that of *Bb*MaeB, in which acetyl-CoA molecules bind to the inside wall of the apex of the triangle formed by the PTA domains (Fig. 2*B*). Comparison of the electrostatic potential surfaces of *Ec*MaeB and *Bb*MaeB shows a significant difference. *Ec*MaeB has an overall negative charge, whereas *Bb*MaeB has an overall positive charge (Fig. 2*C*). Therefore, in *Ec*MaeB, the negative charge prevents negatively charged acetyl-CoA from binding to the inside of the PTA domain hexamer. As a result, acetyl-CoA binds to the positively charged surface outside the PTA domain in *Ec*MaeB. On the other hand, the inside of the PTA domain hexamer of *Bb*MaeB is quite positively charged. Although the outside of the PTA domain is also positively charged, the more positively charged inside wall of the PTA domain in *Bb*MaeB attracts acetyl-CoA.

Comparison of *Ec*MaeB with *Bb*MaeB shows that *Ec*MaeB has bulky amino acids, such as H561, K568, Y573, and Y599 at the site corresponding to the acetyl-CoA binding site of *Bb*MaeB (Fig. S6*A*). At the acetyl-CoA binding site of *Ec*MaeB, the thioacetyl group of acetyl-CoA is inserted into the space created by the α4 and α5 helices and the loop region (V499–R506) (Fig. S6*B*). Meanwhile, a part of the corresponding loop region in *Bb*MaeB (H517–H521) forms a 3_10_-helix, inhibiting accommodation of a ligand molecule.

Comparison of *Ec*MaeB_holo_ and *Ec*MaeB_acetyl-CoA_ shows structural changes in several α helices near the acetyl-CoA binding site (Fig. 2*D*). The α4 helix shifts to approach acetyl-CoA by 1.5 Å and the α5 helix moves away from acetyl-CoA by 1.4 Å. The α3 helix of a nearby protomer (α3′) approaches acetyl-CoA by 2.3 Å. Despite the fact that the binding site of acetyl-CoA in *Bb*MaeB is completely different from that in *Ec*MaeB, shifts of corresponding helices to the same directions are also observed in *Bb*MaeB (Fig. 2*E*).

Due to the ambiguity of the cryo-EM map of the ME domain, detailed discussion on the ME domain structure at an each-residue level is difficult. However, cryo-EM maps for secondary structures show that the ME domain of *Ec*MaeB moves when acetyl-CoA binds to the PTA domain (Fig. S7, *A* and *B*). The movement of the ME domain resulted in a slightly narrower space above the substrate binding site in *Ec*MaeB_acetyl-CoA_ (Fig. S7*C*). This observation suggests the possibility that acetyl-CoA binding makes it difficult for the substrate and/or cofactors to bind to *Ec*MaeB.

### Cryo-EM structures of *Bb*MaeB

**Figure 3:**
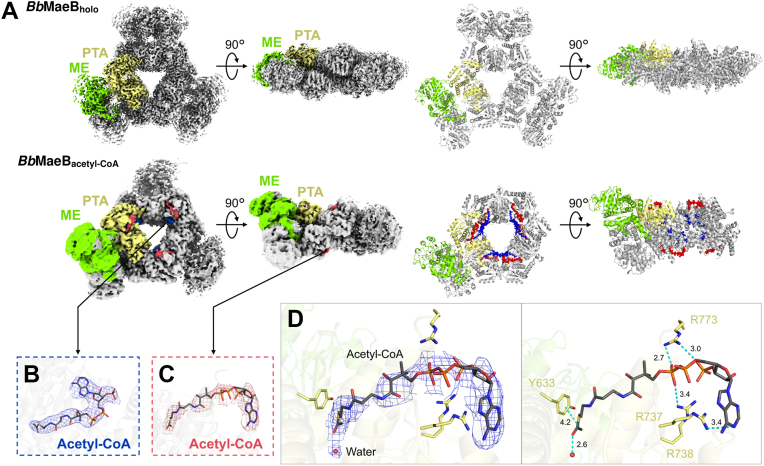
**Cryo-EM structures of *Bb*MaeB.***A*, the cryo-EM map (*left*) and structural model (*right*) of *Bb*MaeB_holo_ and *Bb*MaeB_acetyl-CoA_. The ME domain and the PTA domain are colored *green* and *yellow*, respectively throughout the figure. At the *Bb*MaeB_acetyl-CoA_, acetyl-CoA at the binding site 1 is colored blue and acetyl-CoA at the binding site 2 is *colored red*, respectively throughout the figure. *B* and *C*, the map density of acetyl-CoA at the binding site 1 and 2, respectively. *D*, the EM density of ligands at the binding site 2 of acetyl-CoA in *Bb*MaeB_acetyl-CoA_ (*left panel*). The EM density of acetyl-CoA and water is shown as a mesh, and an acetyl-CoA molecule fit in the density is shown as a stick model colored by elements. Interactions between acetyl-CoA and Y633, R737, R738, and R773 (*right panel*). The numbers next to *dotted lines* indicate the distance between atoms forming an interaction (Å). ME, malic enzyme.

We next determined the structures of *Bb*MaeB using cryo-EM because it is not obvious if the acetyl-CoA binding site previously observed in the crystal structure is the same as that can be observed in the protein dispersed in solution. The cofactors NADP^+^ and Mg^2+^ were added to the protein solution prior to the data collection. First, we collected a cryo-EM data without acetyl-CoA (*Bb*MaeB_holo_). In the result of 2D classification for the *Bb*MaeB_holo_ data, one structure with a fixed orientation of the ME domain corresponding to the H state was dominant (Fig. S4*B*). The structure of *Bb*MaeB_holo_ in the H state was determined at 2.59 Å resolution (Fig. 3*A*). This result is consistent with the previous crystallographic study and confirms that *Bb*MaeB assumes the H state in the absence of acetyl-CoA. Next, we collected data under the condition with acetyl-CoA (*Bb*MaeB_acetyl-CoA_). In the result of 2D classification of the *Bb*MaeB_acetyl-CoA_ data, the ME domain of *Bb*MaeB_acetyl-CoA_ was relatively flexible as was observed in *Ec*MaeB (Fig. S4*C*). We determined the structure of *Bb*MaeB_acetyl-CoA_ at 3.18 Å resolution (Fig. 3*A*). Overall, our cryo-EM experiments reproduce the results from X-ray crystallography that showed acetyl-CoA can induce the structural transition of *Bb*MaeB from the H state to the V state. However, the obtained cryo-EM map of *Bb*MaeB_acetyl-CoA_ shows the *C*1 symmetry and not *D*3 because only one of 3 ME domain dimers clearly shows the V state. The states of the other 2 ME domain dimers are relatively ambiguous, implying incomplete transition from the H state (Fig. S8). In our cryo-EM structure, acetyl-CoA fully occupies all ligand binding sites observed in the *Bb*MaeB crystal structure. Therefore, it is thought that acetyl-CoA binding does not necessarily induce the complete structural change from the H state to the V state.

In the structure of *Bb*MaeB_acetyl-CoA_, acetyl-CoA binds to two binding sites, binding sites 1 and 2 (Fig. 3, *B* and *C*). The binding site 1 is the same as where acetyl-CoA is found in the crystal structure of *Bb*MaeB-HD (Fig. S9*A*). At the binding site 1, acetyl-CoA is stabilized through interactions with R531, K534, R577, and Q679. Importance of these residues on recognition of acetyl-CoA was demonstrated in the earlier study by enzymatic assays with mutant enzymes (53). The binding site 2 is different from the binding site 1. It also differs from the acetyl-CoA binding site of *Ec*MaeB. The binding site 2 is located near the linker helices, which connect the ME and PTA domains and hence are involved in the structural change from the H state to the V state (Fig. S9*B*). Acetyl-CoA at the biding site two is stabilized by hydrogen bonds with R737, R738, and R773, and by hydrophobic interaction with Y633 (Fig. 3*D*). The cryo-EM map of acetyl-CoA at the binding site 2 is less clears than that at the binding site 1, implying that the binding site 1 is the main binding site for acetyl-CoA. Although the binding site 2 is newly discovered in this study, unknown electron densities, which can be interpreted as that of acetyl-CoA, were also observed in the previous crystallographic data of *Bb*MaeB-HD (Fig. S9*C*). This suggests that acetyl-CoA can bind to binding site 2 in *Bb*MaeB-HD as well as in *Bb*MaeB-SSB. The conservation of amino acid sequence of binding site 2 is shown in Fig. S9*D*.

### Detailed structure of acetyl-CoA binding sites in *Ec*MaeB

**Figure 4:**
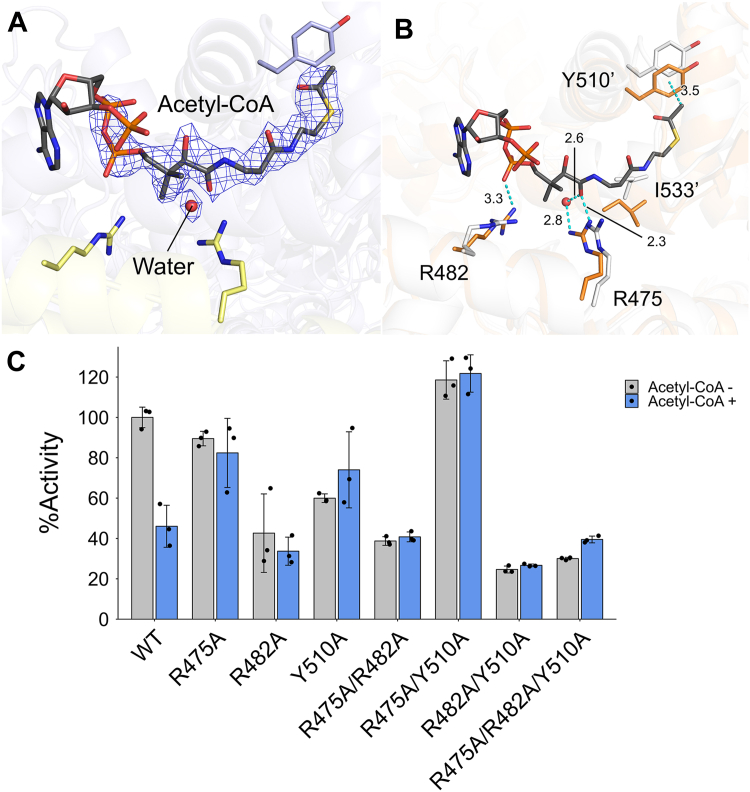
**Acetyl-CoA binding site in *Ec*MaeB.***A*, the EM density of ligands at the binding site of acetyl-CoA molecule that fit in the density is shown as a stick model colored by elements. *B*, structural overlay of *Ec*MaeB_holo_ (*white*) and *Ec*MaeB_acetyl-CoA_ (*orange*) showing changes in residue identity for R475, R482, Y510, and I533 at the binding site of acetyl-CoA in *Ec*MaeB. The numbers next to *dot-dash lines* indicate the distance between atoms forming an interaction (Å). *C*, comparison of the %Activity of *Ec*MaeB WT and variant proteins. *Ec*MaeB mutants are designed to disrupt interactions between acetyl-CoA and R475, R482, and Y510. R475A, Y510A, and R475A/Y510A become insensitive to inhibition by acetyl-CoA. The %Activity was measured by adding the enzyme to a reaction solution containing 5 mM L-malate and 0.5 mM NADP^+^. The final enzyme concentration was adjusted to 1 μM. The allosteric inhibition was observed using a reaction solution containing 5 μM acetyl-CoA.

In the cryo-EM map of *Ec*MaeB_acetyl-CoA_, the adenine ring was almost unobservable (Fig. 4*A*). This result indicates that the adenine ring is not very important in the binding of acetyl-CoA. Acetyl-CoA binds to *Ec*MaeB mainly through interactions with three amino acids: R475, R482, and Y510 (Fig. 4, *A* and *B*). R475 forms a hydrogen bond with one of the carbonyl O atoms of the pantothenic acid moiety in acetyl-CoA. It also indirectly interacts with the same carbonyl O atom through a water molecule. R482 forms a hydrogen bond with one of the phosphate groups of acetyl-CoA. The methyl group of the acetyl tail in acetyl-CoA shows a CH-π interaction with Y510. Comparison of the structure of *Ec*MaeB_holo_ and *Ec*MaeB_acetyl-CoA_ indicates that R475, R482, and Y510 approached acetyl-CoA and that I533 changed the conformation of the side chain.

To confirm the effects of R475, R482, and Y510 on stabilizing acetyl-CoA, we created mutant proteins and measured their activities (Fig. 4*C*, Table S1). The %Activity is defined by the ratio to the activity of the WT enzyme without acetyl-CoA. The WT enzyme showed that the addition of acetyl-CoA reduced the activity by about half under the tested condition. Enzymatic activities of R475A and Y510A mutants were not changed between with and without acetyl-CoA, indicating that they are insensitive to allosteric inhibition by acetyl-CoA. Furthermore, the effect of acetyl-CoA was completely abolished in the R475A/Y510A mutant. These result show that R475 and Y510 play the central role in recognition of acetyl-CoA. In the mutant having R482A (R482A, R475A/R482A, R482A/Y510A, and R475A/R482A/Y510A), the addition of acetyl-CoA did not change their activity, implying that R482 is also involved in stabilization of acetyl-CoA. However, the activities of the R482A mutant series were reduced to about 40% of that of the WT enzyme even in the absence of acetyl-CoA. It is thought that mutation to R482 may cause a conformational change that interferes with the enzymatic activity of *Ec*MaeB.

### Phylogenetic tree of hybrid-type MEs

**Figure 5:**
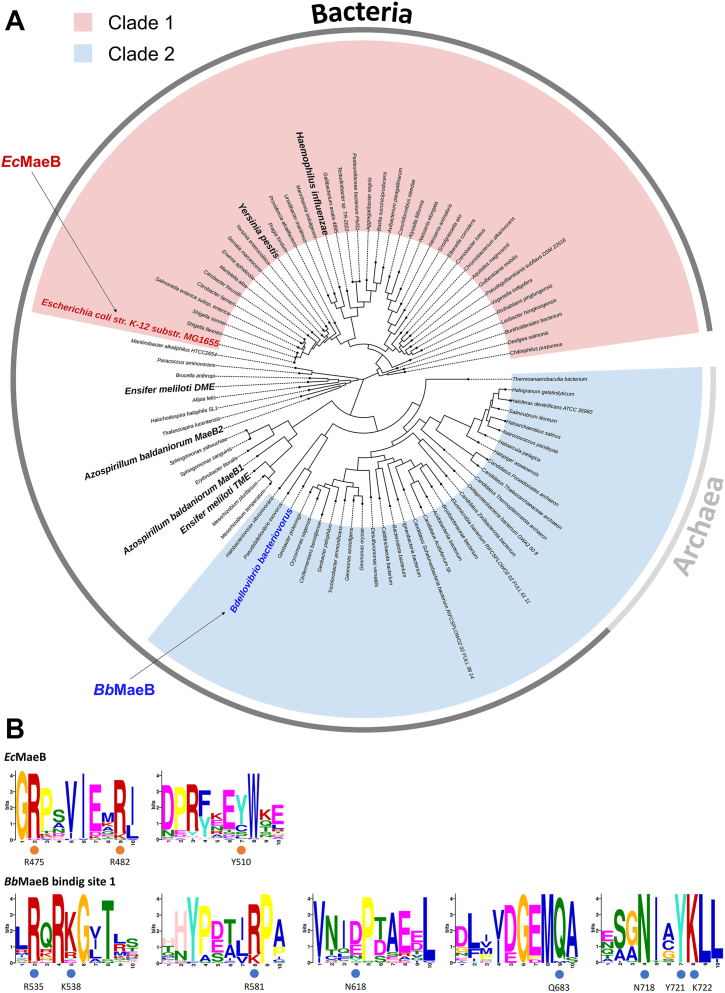
**Classification of prokaryotic hybrid-type MEs.***A*, phylogenetic tree of hybrid-type MEs from various bacteria and archaea. *B*, the acetyl-CoA binding motifs of *Ec*MaeB and *Bb*MaeB binding site 1.

Our enzymatic assay proved that *Ec*MaeB has an effector binding site distinct from that of *Bb*MaeB, meaning that although both *Ec*MaeB and *Bb*MaeB have been classified as the same hybrid-type MEs, they represent different classes of MaeB. We therefore built a phylogenetic tree of hybrid-type MEs of various prokaryotes (Fig. 5*A*). This phylogenetic tree showed that hybrid-type MEs were divided into clade 1, clade 2, and other unclassified clades. Clade 1 contains MEs from well-known pathogenic bacteria such as *Haemophilus influenzae* and *Yersinia pestis* in addition to *E. coli*. On the other hand, *Bb*MaeB is classified as clade 2. Compared to clade 1 hybrid-type MEs, many members of which were from Enterobacteriaceae, clade 2 hybrid-type MEs were distributed among more diverse microorganisms including archaea. Biochemically well-characterized hybrid-type MEs DME, TME, and MaeBs from *Azospirillum baldaniorum* (formerly *Azospirillum brasilense* Sp245) (*Ab*MaeB1 and *Ab*MaeB2) were not classified in either clade 1 or 2. In fact, several amino acids involved in acetyl-CoA binding in *Ec*MaeB and *Bb*MaeB were not conserved in those unclassified bacterial MEs (Figs. S10 and S11). Sequence similarity between *Ec*MaeB and DME or *Ab*MaeB2 and between *Ec*MaeB and TME or *Ab*MaeB1 were about 50 and 30%, respectively. Furthermore, sequence similarity between those four hybrid-type MEs and *Bb*MaeB is low, at about 30%. These results suggest that MEs from *Ensifer. meliloti* and *A. baldaniorum* have evolved independently from hybrid-type MEs belonging to clades 1 and 2.

In both clades 1 and 2, the amino acids involved in acetyl-CoA binding were conserved (Figs. 5*B* and S9*D*). In *Ec*MaeB, R475 and R482 were highly conserved whereas Y510 was often replaced by cysteine, serine and leucine. At the binding site 1 of *Bb*MaeB, many amino acids involved in acetyl-CoA binding were conserved. N618 was poorly conserved, but it is usually replaced with aspartate and glutamate, which can form similar interaction. Within binding site 2 of *Bb*MaeB, the amino acids that mediate acetyl-CoA binding exhibit lower conservation. Notably, Y633 is hardly conserved; however, it is often replaced by phenylalanine or leucine that could similarly engage acetyl-CoA through hydrophobic interactions. In addition, the residue at position R738, which interacts with the phosphate group of acetyl-CoA, is typically maintained as a positively charged arginine or lysine in other clade 2 MEs, whereas the residue at R737 is considerably less conserved.

## Discussion

In this study, we determined the structures of *Ec*MaeB and *Bb*MaeB, providing insights into the bacterial hybrid-type MEs. Earlier reports show that acetyl-CoA decreases the activity of these enzymes and that the PTA domain is involved in the allosteric regulation. Our structural and enzymatic experiments demonstrated that *Ec*MaeB and *Bb*MaeB have distinct effector binding sites in the PTA domain; however, binding of acetyl-CoA causes the same structural changes at the α4, α5, and α3′ helices, indicating that acetyl-CoA regulates the activities of these enzymes depending on the same mechanism.

**Figure 6:**
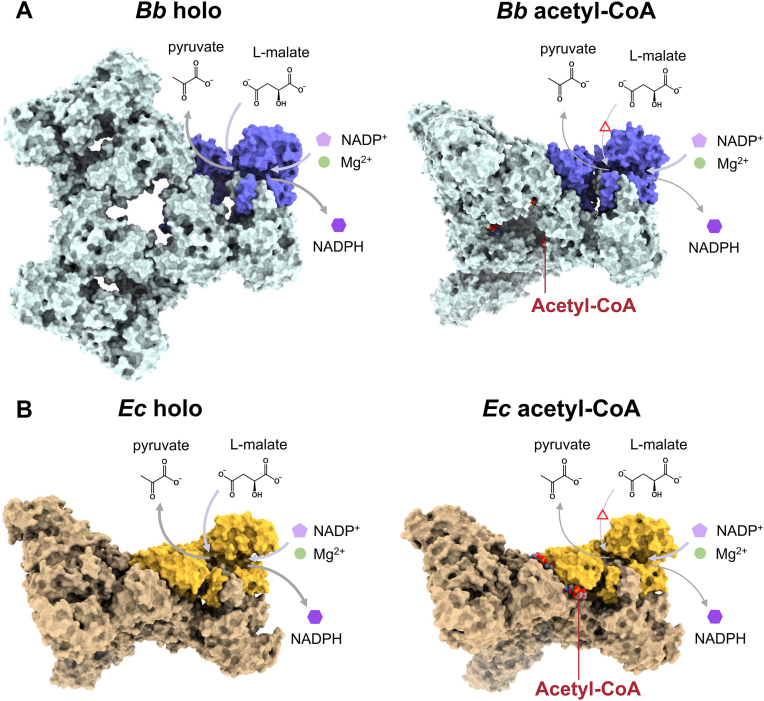
**Schematic diagram of the enzymatic reaction and allosteric regulation by acetyl-CoA.***A*) *Bb*MaeB_holo_ (*left*) and *Bb*MaeB_acetyl-CoA_ (*right*). In *Bb*MaeB_acetyl-CoA_, the dynamic conformational changes in the ME domain restrict the entry of the substrate, L-malate, into the catalytic site. *B*, *Ec*MaeB_holo_ (*left*) and *Ec*MaeB_acetyl-CoA_ (*right*). In *Ec*MaeB_acetyl-CoA_, narrowing of the space near the catalytic site restricts entry into the catalytic site.

The earlier crystallographic study proposed that acetyl-CoA binding controls the activity of *Bb*MaeB through the dynamic structural transition from the H state to the V state. Our cryo-EM data also shows that acetyl-CoA induces the similar movement of the ME domain although the transition to the V state in cryo-EM data is less obvious than in the crystallographic data. In contrast, the crystal and cryo-EM structures of *Ec*MaeB only show the V state both in the presence and absence of acetyl-CoA. Activity assay of our present study and earlier reports show that compared to *Ec*MaeB, *Bb*MaeB is greatly inhibited by low concentrated acetyl-CoA, which suggests that *Bb*MaeB is more sensitive to acetyl-CoA than *Ec*MaeB. It is possible that the sensitivity to acetyl-CoA is related to the clarity of reorientation of the ME domain. Moreover, we found that *Bb*MaeB has two acetyl-CoA binding sites on the PTA domain. Because mutations to the amino acids at the binding site 1 in *Bb*MaeB-HD eliminates the allosteric effect (53), the binding site 1 seems to be enough to trigger the allosteric regulation. The role of the binding site 2 is therefore unknown, but it may support the transition from the H state to the V state because it is located near the linker helices that are involved in the structural changes. In *Bb*MaeB, the change of the positions of ME domains on binding of acetyl-CoA leads to partially blocking the channel through which substrate approaches the catalytic site (Fig. 6*A*). Likewise, binding of acetyl-CoA and consequent structural changes make the space above the catalytic site in *Ec*MaeB narrower (Fig. 6*B*). Thus, in both MaeBs, acetyl-CoA binding appears to hinder substrate access to the catalytic site. In this model, the reduced catalytic activity of hybrid-type MEs can be attributed to decreased accessibility rather than direct alteration of the active site. As the local structure of the catalytic center remains largely unchanged, substrate affinity is expected to remain unaffected, which aligns with the observed kinetic data.

Phylogenetic analysis revealed that hybrid-type MEs of microorganisms are classified into clade 1, clade 2, and others. *Ec*MaeB is a typical clade 1 ME, while *Bb*MaeB a typical clade 2 ME. The acetyl-CoA binding sites are well conserved only within each clade. On one hand, in clade 1, the site corresponding to the acetyl-CoA binding site 1 of clade 2 contains bulky amino acids that inhibit the binding of acetyl-CoA. On the other hand, in clade 2, the site corresponding to the acetyl-CoA binding site of clade 1 has a secondary structure that prevents acetyl-CoA binding. These observations indicate that the allosteric effector binding site of hybrid-type MEs in clades 1 and 2 have evolved in a mutually exclusive manner. Nevertheless, these allosteric inhibitor binding sites are modulated through convergent evolution in order to trigger similar structural changes.

In the phylogenetic analysis, we observed the presence of hybrid-type MEs that do not belong to either clade 1 or 2. Among them are DME, TME, and *Ab*MaeBs, which are biochemically well-characterized. These enzymes are known to show responses to acetyl-CoA different from *Ec*MaeB and *Bb*MaeB. The activity of *Ab*MaeB1 is regulated by acetyl-CoA but it requires higher concentrated acetyl-CoA to show allosteric inhibition compared to *Ec*MaeB and *Bb*MaeB (54). Moreover, response to the acetyl-CoA/CoA ratio is different between *Ab*MaeB1 and *Ec*MaeB. While DME is inhibited by acetyl-CoA, propionyl-CoA, and butyryl-CoA, TME does not show any allosteric regulation by CoA-containing chemicals including acetyl-CoA (55). In these unclassified clades, the amino acid residues of the acetyl-CoA binding sites in *Ec*MaeB and *Bb*MaeB were not well conserved. Therefore, they may have evolved independently to construct acetyl-CoA binding sites that differ from those of *Ec*MaeB and *Bb*MaeB. Future structural studies of unclassified hybrid-type MEs will help to know their molecular mechanism of allosteric regulation.

In general, allosteric sites have become more complex as prokaryotes have evolved into eukaryotes. For example, eukaryotic ATP-dependent 6-phospho-fructokinase and glutamate dehydrogenase exhibit much more complex allosteric regulatory mechanisms than their prokaryotic counterparts (56, 57). However, the amino acid sequence of the allosteric site to which the same effector ligand binds is usually conserved among the same enzymes even from phylogenetically distant organisms. Recently, drug discovery research focusing on allosteric sites has been active because in terms of drug design, the allosteric site is superior to the active site, which is the classical drug-target pocket (58). In addition, allosteric sites have the potential to discover not only inhibitors but also activators (59, 60). Here, we provide an example of “the same effector molecule binding to different binding sites in the same enzyme with the same allosteric effect”, which opens up the possibility of future discoveries of similar phenomena in other proteins. For example, an inhibitor compound that binds to a protein of a model organism can also bind to a human homologous protein but at a different site. In such a case, the compound can show a similar allosteric effect; however, if the structure of the drug candidate is tuned based on the model organism protein, it no longer has the same effect in humans.

## Experimental procedures

### Sequence and phylogenetic analyses

Protein sequences of interest were obtained from the database of The National Center for Biotechnology Information (https://www.ncbi.nlm.nih.gov). Amino acid sequence analyses were performed by MEGA X (61). BLAST searches were performed *via* the web service for protein blast (https://blast.ncbi.nlm.nih.gov/Blast.cgi) (62). Phylogenetic analysis of the hybrid-type MEs was performed by using only PTA domains using the MEGA X implementation of MUSCLE. A maximum likelihood method was used to estimate a phylogenetic tree. A figure of the phylogenetic tree was illustrated by using iTOL v6 (https://itol.embl.de/) (63). The list of the species for the phylogenetic analysis and GenBank accession numbers are provided in Table S2. Figures of sequence alignments were generated by ESPript 3.0 (http://espript.ibcp.fr) (64). Figures of binding motifs were visualized by MEME Suite 5.5.7 (https://meme-suite.org/meme/) (65).

### Protein expression and purification

To obtain the *E. coli* MaeB gene, we used *E. coli* strain SHuffle T7 because the amino acid sequence of MaeB of this strain is the same as that of *E. coli* strain K12 (UniProt entry ID: P76558). The genomic DNA of *E. coli* strain SHuffle T7 was purified with NucleoSpin Microbial DNA (Macherey-Nagel, Düren, Germany) according to the instruction manual. The gene of *Ec*MaeB was amplified from the *E. coli* genomic DNA by PCR with primers shown in Table S3. The synthesized gene (gBlocks Gene Fragment) of *B. bacteriovorus* strain SSB218315 MaeB (*Bb*MaeB-SSB; UniProt entry ID: A0A1Z3N8R4) was purchased from Integrated DNA Technologies (Coralville, IA, USA). For high sequence accuracy, the full-length DNA sequence of *Bb*MaeB-SSB was separated into two parts, *Bb*MaeB-SSB-1 and *Bb*MaeB-SSB-2. The MaeB genes were cloned in the pET-28a(+) vector (Novagen). A pET-28a(+) plasmid containing the *Ec*MaeB gene with an C-terminal His-tag and a pET-28a(+) plasmid containing the *Bb*MaeB gene with an N-terminal His-tag were transformed to *E.coli* expression strain BL21(DE3) cells and grown in LB media containing 100 μg/ml kanamycin at 37 °C. Once the cell reached 0.6 OD_600_, expression was induced with 0.5 mM IPTG and the temperature was reduced to 18 °C for approximately 18 h. Cells were harvested by centrifugation for 30 min at 6000×*g*. The supernatant was discarded and the pellets were stored at −80 °C. Pellets were resuspended into Buffer A (50 mM Hepes pH 7.5, 300 mM NaCl, 5 mM imidazole, a cOmplete Protease Inhibitor Cocktail tablet (Roche)). Cells were lysed using sonication. Insoluble cell debris was removed by centrifugation for 1 h at 20,000×*g*. The soluble material was passed over a HisTrap TALON column (Cytiva), pre-equilibrated with Buffer A. The column was washed with 7 column volumes (CV) of Buffer A to remove non-specific interacting proteins. Then to remove co-purifying CoA and acetyl-CoA, the column was washed with 20 CV of a mild denaturing buffer (2.5 M Urea, 2.5 M KBr, 50 mM Hepes pH 7.5). Another 10 CV of lysis buffer was passed over the column to remove the denaturing agents before the column was eluted with 7 CV of Buffer B (50 mM Hepes pH 7.5, 300 mM NaCl, 200 mM Imidazole). Pooled fractions were transferred to 2 kDa MWCO dialysis tubing and dialyzed against 5 L of dialysis buffer (50 mM Hepes pH 7.5, 300 mM NaCl) for 18 h at 4 °C. After dialysis, protein solution was purified by size-exclusion chromatography on a Hiload 16/60 Superdex 200 prep grade column (Cytiva), using Buffer C (50 mM Hepes pH 7.5, 300 mM NaCl). Fractions of size-exclusion chromatography were collected, concentrated, aliquoted, and kept at −80 °C.

Creation of mutant proteins (R475A, R482A, Y510A, R475A/R482A, R475A/Y510A, R482A/Y510A, and R475A/R482A/Y510A) was performed using KOD -Plus- Mutagenesis kit (TOYOBO) according to the instruction manual. The primers sequencing for cloning and mutagenesis are provided in Table S3. Constructs and mutations were confirmed by sequencing, before transformation into the *E. coli* expression strain BL21(DE3).

### Crystallization

Crystallization was performed by the sitting drop and hanging drop vapor-diffusion method. Crystals of *Ec*MaeB appeared under the condition of 20 mg/ml protein, 0.2 M Potassium chloride pH 7.0, 20% v/v Polyethylene glycol 3350 at 20 °C. Before the crystals were frozen by liquid nitrogen, they were soaked in the crystallization solutions supplemented by 15% v/v Ethylene glycol.

### X-ray data collection, processing, structure solution, and refinement

X-ray diffraction experiment was performed on the BL44XU beamline of SPring-8. Diffraction images were collected at 100 K using an EIGER X 16 M detector (Dectris). For data collection, X-ray wavelengths were set to 0.9 Å, respectively. The datasets were processed using XDS (66). The processed data were scaled and merged by Aimless (67). Phase determination and initial model building was performed by Molrep (68). Manual model building was performed using Coot (69). The program Refmac5 (70) and phenix.refine (71) were used for structural refinement. The stereochemical quality of the final model was checked by Molprobity (72). Data collection and refinement statistics are summarized in Table S4.

### Cryo-EM specimen preparation

*Ec*MaeB and *Bb*MaeB were diluted to a final concentration of 0.5 mg/ml. NADP^+^ and MgCl_2_ were added to the samples at final concentrations of 1 mM and 3 mM, respectively. In *Ec*MaeB_acetyl-CoA_ and *Bb*MaeB_acetyl-CoA_, acetyl-CoA was added to achieve a final concentration of 60 μM. A total of 3 μl of the complex solutions was applied onto a freshly glow-discharged QUANTIFOIL R1.2/1.3 Cu 200 grids (Quantifoil Micro Tools GmbH) and then blotted for 3 s before rapidly cryocooling them in liquid ethane. For blotting and freezing the grids, a Vitrobot Mark IV (Thermo Fisher Scientific) device was used, and the sample chamber was kept at 8 °C and 100% humidity.

### Cryo-EM data collection

For the high-resolution study, *Ec*MaeB_holo_, *Bb*MaeB_holo_, and *Bb*MaeB_acetyl-CoA_ were imaged in CRYO ARM 200 (JEOL) electron microscope at 200 kV, with a Gatan K3 direct electron detector controlled by SerialEM (73) at the calibrated magnification of × 60,000, yielding a pixel size of 0.83 Å. *Ec*MaeB_acetyl-CoA_ was imaged in CRYO ARM 300 (JEOL) electron microscope at 300 kV, with a Gatan K3 direct electron detector at the calibrated magnification of × 60,000, yielding a pixel size of 0.87 Å.

For the first dataset and the second dataset, a total dose of 40.00  electrons  Å^−2^ was fractionated over 40 frames. The defocus targets were −0.7 to −2.2 μm for the first dataset and −0.7 to −2.0 μm for the second dataset.

### Image processing

Data processing was performed in CryoSPARC (74). For the *Ec*MaeB_holo_ and *Ec*MaeB_holo-ME_ dataset, Motion correction, contrast transfer function (CTF) estimation, particle picking, 2D classification, and further data processing were performed with CryoSPARC. An initial set of particles was automatically picked using Blob picker. The extracted particles were used for Template picker and further 2D classification. Then, an *Ab*-*initio* reconstruction was generated after representative particles were selected. Heterogeneous refinement and homogeneous refinement were performed to isolate particles contributing to the best reconstructions of *Ec*MaeB_holo_ after *Ab*-*initio* model was generated. After that, 127,548 particles were subjected to CTF refinement and Non-uniform refinement with a *D*3 symmetry. In addition, Local refinement of the ME domain dimer was performed for the observation of cofactors. The detail of the workflow for cryo-EM data processing of *Ec*MaeB_holo_ and *Ec*MaeB_holo-ME_ is shown in Fig. S12.

For the *Ec*MaeB_acetyl-CoA_ and *Ec*MaeB_acetyl-CoA-ME_ dataset, Motion correction, CTF estimation, particle picking, 2D classification, and further data processing were performed with CryoSPARC. An initial set of particles was automatically picked using Blob picker. The extracted particles were used for Template picker and further 2D classification. Then, Homogeneous refinement and Heterogeneous refinement were performed to isolate particles contributing to the best reconstructions of *Ec*MaeB_acetyl-CoA_. After that, 531,529 particles were subjected to CTF refinement, Non-uniform refinement, and Reference based motion correction with a *D*3 symmetry. In addition, Local refinement of the ME domain dimer was performed for the observation of cofactors. The detail of the workflow for cryo-EM data processing of *Ec*MaeB_acetyl-CoA_ and *Ec*MaeB_acetyl-CoA-ME_ is shown in Fig. S13.

For the *Bb*MaeB_holo_ dataset, Motion correction, CTF estimation, particle picking, 2D classification, and further data processing were performed with CryoSPARC. An initial set of particles was automatically picked using Blob picker. The extracted particles were used for Template picker and further 2D classification. Then, an *Ab*-*initio* reconstruction was generated after representative particles were selected. Homogeneous refinement was performed to isolate particles contributing to the best reconstructions of *Bb*MaeB_holo_ after *Ab*-*initio* model was generated. After that, 449,832 particles were subjected to CTF refinement, Non-uniform refinement, and Reference based motion correction with a *D*3 symmetry. The detail of the workflow for cryo-EM data processing of *Bb*MaeB_holo_ is shown in Fig. S14.

For the *Bb*MaeB_acetyl-CoA_ dataset, Motion correction, CTF estimation, particle picking, 2D classification, and further data processing were performed with CryoSPARC. An initial set of particles was automatically picked using Blob picker. The extracted particles were used for Template picker and further 2D classification. Then, an *Ab*-*initio* reconstruction was generated after representative particles were selected. Homogeneous refinement and Heterogeneous refinement were performed to isolate particles contributing to the best reconstructions of *Bb*MaeB_acetyl-CoA_ after *Ab*-*initio* model was generated. After that, 165,921 particles were subjected to CTF refinement and Nonuniform refinement with a *C*1 symmetry. The detail of the workflow for cryo-EM data processing of *Bb*MaeB_acetyl-CoA_ is shown in Fig. S15.

Cryo-EM data processing yielded 2.73, 3.19, 2.03, 2.39, 2.59, and 3.18 Å maps for the *Ec*MaeB_holo_, *Ec*MaeB_holo-ME_, *Ec*MaeB_acetyl-CoA_, *Ec*MaeB_acetyl-CoA-ME_, *Bb*MaeB_holo_, and *Bb*MaeB_acetyl-CoA_, respectively. These resolutions for the final maps were estimated by the 0.143 criterion of the Fourier shell correlation curve.

### Model building

The atomic models of *Ec*MaeB and *Bb*MaeB were built from the composite map. The crystal structure of *Ec*MaeB_apo_ (PDB code ID: 9KRW) was used to initial template for model building of *Ec*MaeB_holo_. Then, the structure of *Ec*MaeB_holo_ was used to generate initial templates for model building of *Ec*MaeB_acetyl-CoA_. An AlphaFold predicted model (75) and the crystal structure of *Bb*MaeB acetyl-CoA bound form (PDB code ID: 6ZNG) were used to initial templates for model building of *Bb*MaeB_holo_ and *Bb*MaeB_acetyl-CoA_, respectively. Each of *Ec*MaeB and *Bb*MaeB was fitted into the composite EM density maps using UCSF chimera (76) and then manually adjusted, built and real space refined using Coot (69) and “real-space refinement (77)” in Phenix (78). Water molecules were modeled only in the ligand periphery. All structural figures are visualized and generated by UCSF ChimeraX (79) or PyMOL (https://www.pymol.org/). The statistics for all data collection and structure refinement are summarized in Tables S5 and S6.

### Enzyme assay and inhibitor studies

Spectrophotometric assays were used to monitor the oxidative decarboxylation of L-malate to pyruvate using a SpectraMax M2, UV-Visible spectrophotometer (Molecular Devices) operated by SoftMax Pro Software with kinetic mode. All assays recorded the Δabsorbance at 340 nm (NADPH) at 30 °C. Assays were initiated by the addition of enzyme to the otherwise complete reaction mixture. The effects of ME effector molecules and *Ec*MaeB mutants were tested against *Ec*MaeB activity under standard reaction conditions: 100 mM Hepes-NaOH pH 7.5, 50 mM NaCl, 5 mM MgCl_2_, 5 mM L-malate, 0.5 mM NADP^+^ and 1 μM enzyme. Acetyl-CoA was tested at 5 μM. The results are presented as “%Activity”: a percentage of the measured activity in the presence of effector compared to the measured activity of MaeB under standard reaction conditions.

Enzyme activity parameters were determined by varying the concentrations of L-malate and NADP^+^, respectively. The assay was performed under the standard reaction condition as described above, varying either L-malate or NADP^+^ concentration. Final concentration of 100 nM enzyme was used, except for Michaelis-Menten plots and empirical Hill plots for MaeBs with or without acetyl-CoA (Fig. S1*E*), which we used 300 nM enzyme due to the lower activity.

Data with fixed malate concentrations were fitted to the standard Michaelis-Menten model: *v*_0_ = *V*_max_ [NADP^+^]/(*K*_m_ + [NADP^+^]), where *v*_0_, *V*_max_, and *K*_m_ indicate the reaction velocity, the maximal velocity, and the Michaelis constant, respectively. Data with fixed NADP^+^ concentrations were fitted to the substrate inhibition model described by LiCata and Allewell (80):

$$v_{0}=\frac{V_{\max}+V_{i}\cdot \left(\frac{{\left[\text{malate}\right]}^{x}}{K_{i}^{x}}\right)}{1+\left(\frac{K_{m}^{h}}{{\left[\text{malate}\right]}^{h}}\right)+\left(\frac{{\left[\text{malate}\right]}^{x}}{K_{i}^{x}}\right)}$$

where *V*_i_, *K*_i_, *h*, and *x* indicate the reaction velocity in the presence of inhibition, the inhibition constant, the Hill coefficient, and a second Hill coefficient, respectively. For this substrate inhibition model, *x* should be fixed. In our fitting, it was set to 10 because using *x* < 10 failed to converge to realistic parameter values. The plots shown in Fig. S1*F* were fitted to the empirical Hill equation:

$$v_{0}=V_{\max}\cdot {\left[\text{malate}\right]}^{h}/\left(K_{0.5}^{h}+{\left[\text{malate}\right]}^{h}\right)$$

where *K*_0.5_ indicates the substrate concentration for half-saturation. When *h* is equal to 1, *K*_0.5_ matches Michaelis constant *K*_m_.

The IC_50_ plot for Acetyl-CoA against MaeB activity was obtained by varying acetyl-CoA concentrations. The assay was performed in the standard reaction condition with 100 nM final enzyme concentration. The data were fitted to the four-parameter logistic model:

$$v_{0}=\text{Bottom}+\frac{\text{Top}-\text{Bottom}}{1+{\left(\frac{\left[\text{Acetyl}-\text{CoA}\right]}{{\text{IC}}_{50}}\right)}^{h}}$$

where *h* means the Hill slope value and “Top” and “Bottom” corresponds to the maximum and minimum *v*_0_ of the fitted curve, respectively (81). Fitting was performed using Rstudio.

## Data availability

The atomic coordinates and structure factors of *Ec*MaeB_apo_ have been deposited in the Protein Data Bank (PDB) with PDB accession code 9KRW. The cryo-EM density maps and the atomic coordinates of *Ec*MaeB_holo_, *Ec*MaeB_holo-ME_, *Ec*MaeB_acetyl-CoA_, *Ec*MaeB_acetyl-CoA-ME_, *Bb*MaeB_holo_, and *Bb*MaeB_acetyl-CoA_ have been deposited in the Electron Microscopy Data Bank (EMDB) and the PDB with EMDB accession codes EMD-62540, EMD-63586, EMD-62816, EMD-63599, EMD-62541, and EMD-62542 and PDB accession codes 9KRT, 9M2I, 9L4N, 9M35, 9KRU, and 9KRV, respectively. The raw images of *Ec*MaeB_holo_ and *Ec*MaeB_holo-ME_, *Ec*MaeB_acetyl-CoA_ and *Ec*MaeB_acetyl-CoA-ME_, *Bb*MaeB_holo_, and *Bb*MaeB_acetyl-CoA_ have been deposited in the Electron Microscopy Public Image Archive (EMPIAR) (82) with EMPIAR accession codes EMPIAR-12691, EMPIAR-12692, EMPIAR-12693, and EMPIAR-12694, respectively.

## Supporting information

This article contains supporting information.

## Conflict of interest

The authors declare that they have no conflicts of interest with the contents of this article.
