# Supplementary material for: Divergent acetyl-CoA binding modes mediate allosteric inhibition of bacterial hybrid-type malic enzymes
Source: J Biol Chem. 2025 Nov 4;301(12):110887. doi: 10.1016/j.jbc.2025.110887 (PMC12704298; doi:10.1016/j.jbc.2025.110887)
Supplement: Supporting Tables and Figures [file mmc1.docx]

**Supporting Information for**

**Divergent acetyl-CoA binding modes mediate allosteric inhibition of bacterial hybrid-type malic enzymes**

**Authors:** Munetoshi Sassa, Haruka Yamato, Hiroki Tanino, Yohta Fukuda, Tsuyoshi Inoue

**1. Tables (pp. 2–8)**

Table S1. Enzymatic activity of *Ec*MaeB wild-type and variant proteins.

Table S2. The list of the species for the phylogenetic analysis and GenBank accession numbers.

Table S3. Primer sequences used in this research.

Table S4. X-ray crystallography data collection, processing, and refinement parameters.

Table S5. Cryo-EM data collection parameters and model statistics of *Ec*MaeB.

Table S6. Cryo-EM data collection parameters and model statistics of *Bb*MaeB.

**2. Figures (pp. 9–23)**

Fig. S1. Enzymatic assays for *Ec*MaeB and *Bb*MaeB.

Fig. S2. Representative electron density maps of the *Ec*MaeB crystal structure.

Fig. S3. Detailed description of the *Ec*MaeB crystal structure.

Fig. S4. Representative particles after 2D classification.

Fig. S5. The cryo-EM maps of cofactors.

Fig. S6. Comparison of acetyl-CoA binding sites between *Ec*MaeB and *Bb*MaeB.

Fig. S7. Structural comparison of the ME domain dimers *Ec*MaeB.

Fig. S8. Difference of the conformation of the ME domain dimers in *Bb*MaeB_acetyl-CoA_.

Fig. S9. Acetyl-CoA binding sites of *Bb*MaeB.

Fig. S10. Sequence alignment of hybrid-type MEs from an unclassified clade near clade 1.

Fig. S11. Sequence alignment of hybrid-type MEs from an unclassified clade near clade 2.

Fig. S12. Cryo-EM analysis of *Ec*MaeB_holo_ and *Ec*MaeB_holo-ME_.

Fig. S13. Cryo-EM analysis of *Ec*MaeB_acetyl-CoA_ and *Ec*MaeB_acetyl-CoA-ME_.

Fig. S14. Cryo-EM analysis of *Bb*MaeB_holo_.

Fig. S15. Cryo-EM analysis of *Bb*MaeB_acetyl-CoA_.

**Tables**

**Table S1 | Enzymatic activity of *Ec*MaeB wild-type and variant proteins.**

| variants | **Without Acetyl-CoA** | | **With Acetyl-CoA** | |
| --- | --- | --- | --- | --- |
|  | **NADP^+^ production**  **(µM·min^-1^)** | **Activity**  **(%)** | **NADP^+^ production**  **(µM·min^-1^)** | **Activity**  **(%)** |
| WT | 26.2 ± 1.1 | 100 ± 4.2 | 12.1 ± 2.2 | 46.4 ± 8.5 |
| R475A | 23.4 ± 0.8 | 89.5 ± 2.9 | 21.6 ± 3.7 | 82.4 ± 14.0 |
| R482A | 11.2 ± 4.2 | 42.6 ± 15.9 | 8.4 ± 1.4 | 32.3 ± 5.5 |
| Y510A | 15.7 ± 0.5 | 60.0 ± 1.7 | 19.4 ± 4.0 | 74.0 ± 15.4 |
| R475A/R482A | 10.2 ± 0.5 | 38.7 ± 1.7 | 10.7 ± 0.5 | 40.8 ± 2.0 |
| R475A/Y510A | 31.1 ± 2.0 | 118.6 ± 7.8 | 31.9 ± 2.0 | 121.9 ± 7.6 |
| R482A/Y510A | 6.4 ± 0.4 | 24.6 ± 1.4 | 7.0 ± 0.1 | 26.7 ± 0.5 |
| R475A/R482A/Y510A | 7.9 ± 0.1 | 30.0 ± 0.5 | 10.3 ± 0.4 | 39.5 ± 1.4 |

**Table S2 | The list of the species for the phylogenetic analysis and GenBank accession numbers.**

| **Taxon name** | **GenBank accession** | **Taxon name** | **GenBank accession** |
| --- | --- | --- | --- |
| *E. coli* str. K-12 substr. MG1655 | AAC75516.1 | *E. litoralis* | KEO91005.1 |
| *S. sonnei* | EIH1816076.1 | *M. temperatum* | PAQ07877.1 |
| *S. flexneri* | HBD4497155.1 | *M. plurifarium* | CDX57256.1 |
| *C. farmeri* | WP_195297197.1 | *S. sanguinis* | KTT68362.1 |
| *C. freundii* | AOI30470.1 | *S. yanoikuyae* | ATI80926.1 |
| *S. enterica* subsp. enterica | EEJ6667587.1 | *T. lucentensis* | KZB67244.1 |
| *G. mobilis* | WP_054285865.1 | *H. halophila* SL1 | ABM61805.1 |
| *C. alkanivorans* | MBN3006691.1 | *P. aminovorans* | SFH07682.1 |
| *A. magnusonii* | PXX48970.1 | *M. alkaliphilus* HTCC2654 | EAQ15065.1 |
| *Crenobacter luteus* | WP_066612193.1 | *A. felis* | CEG08232.1 |
| *P. subflava* DSM 22618 | SMF56171.1 | *B. anthropi* | KAB2773182.1 |
| *V. indigofera* | WP_272766087.1 | *B. bacteriovorus* | ASD63860.1 |
| *R. pingtungensis* | WP_312264643.1 | *P. exovorus* | WP_015469788.1 |
| *L. hongkongensis* | WP_193583283.1 | *Ignavibacteria bacterium* | MDH7526816.1 |
| *Burkholderiales bacterium* | MBV8467622.1 | *O. sagensis* | WP_151157012.1 |
| *E. corrodens* | WP_064105400.1 | *G. oryzae* | WP_129125841.1 |
| *S. alvi* | PIT37432.1 | *G. azotofigens* | WP_216512085.1 |
| *N. animaloris* | MDO5072812.1 | *G. pickeringii* | WP_039742007.1 |
| *N. elongata* | WP_040665771.1 | *Elusimicrobia bacterium* RIFCSPLOWO2_02_FULL_61_11 | OGS04597.1 |
| *C. steedae* | WP_124793885.1 | *H. vibrionivorans* | RZF22181.1 |
| *D. salmonis* | WP_226764431.1 | *C. bemidjiense* | WP_012529886.1 |
| *C. purpureus* | WP_263126579.1 | *D. versatilis* | WP_221252247.1 |
| *M. alba* | WP_136989985.1 | *C. Schekmanbacteria bacterium* RIFCSPLOWO2_02_FULL_38_14 | OGL51708.1 |
| *A. filiformis* | WP_097113530.1 | *Bryobacteraceae bacterium* | HSB18128.1 |
| *P. alcalifaciens* | WP_225575468.1 | *Acidobacteriia bacterium* | MCL6565602.1 |
| *S. marcescens* | BEN22046.1 | *T. ammonificans* | WP_305731230.1 |
| *Y. enterocolitica* | WP_050138797.1 | *G. pelophilus* | MBT0665861.1 |
| *P. fontium* | WP_074824810.1 | *B. bacterium* | MDI6803965.1 |
| *Testudinibacter* sp. TR-2022 | WP_139613899.1 | *C. bacterium* | NOZ60328.1 |
| *E. aphidicola* | WP_230049385.1 | *C. Acidoferrum* sp. | HMD44139.1 |
| *U. maritimus* | WP_157403199.1 | *Deltaproteobacteria bacterium* GWA2_50_8 | OGP14631.1 |
| *H. influenzae* | WP_105880104.1 | *C. Zixiibacteriota bacterium* | MEW5874260.1 |
| *B. succiniciproducens* | WP_100052073.1 | *Thermoanaerobaculia bacterium* | MEE8525570.1 |
| *A. paragallinarum* | WP_194752046.1 | *C. Thermoplasmatota archaeon* | HET6403914.1 |
| *M. indoligenes* | WP_334235829.1 | *H. gelatinilyticum* | WP_089696784.1 |
| *G. anatis* 4895 | KGQ60508.1 | *H. salinus* | WP_256297126.1 |
| *A. segnis* | WP_315027877.1 | *H. pelagica* | WP_284008843.1 |
| *Pasteurellaceae bacterium* Phil31 | TNH04919.1 | *S. litoreum* | WP_227227949.1 |
| *Y. pestis* | WP_087794156.1 | *H. aswanensis* | WP_120246796.1 |
| *A. baldaniorum* MaeB1 | WP_014238784.1 | *N. pandeyae* | WP_148857492.1 |
| *A. baldaniorum* MaeB2 | WP_014198543.1 | *H. denitrificans* ATCC 35960 | EMA04190.1 |
| *E. meliloti* DME | AAB82459.1 | *C. Poseidoniales archaeon* | RCH74090.1 |
| *E. meliloti* TME | AAB82460.1 | *C. Thalassarchaeaceae archaeon* | MDG1540612.1 |

**Table S3 | Primer sequences used in this research.**

| **Construct** |  | **Primer Sequence** |
| --- | --- | --- |
| *Ec*MaeB | Forward | 5’ AGAAGGAGATATACCATGGATGACCAGTTAAAACAAAGTGCACTTG 3’ |
|  | Reverse | 5’ GTGGTGGTGGTGGTGCAGCGGTTGGGTTTGCGC 3’ |
| *Ec*MaeB  R475A | Forward | 5’ GGTGCGCCGAACGTGATCGAAATGC 3’ |
|  | Reverse | 5’ GATAAGGATCGGTTTCGCCAGTCCC 3’ |
| *Ec*MaeB  R482A | Forward | 5’ ATGGCGATTCAGAAACTGGGCTTGC 3’ |
|  | Reverse | 5’ TTCGATCACGTTCGGACGACCGATA 3’ |
| *Ec*MaeB  Y510A | Forward | 5’ GAGGCGTGGACCGAATACTTCCAGA 3’ |
|  | Reverse | 5’ TTTAAAGCGCGGATCGGATTCGTTA 3’ |
| *Ec*MaeB  R475A/R482A | Forward | 5’ ATGGCGATTCAGAAACTGGGCTTGC 3’ |
|  | Reverse | 5’ TTCGATCACGTTCGGCGCACCGATA 3’ |
| *Ec*MaeB  R475A/Y510A | Forward | 5’ GGTGCGCCGAACGTGATCGAAATGC 3’ |
|  | Reverse | 5’ GATAAGGATCGGTTTCGCCAGTCCC 3’ |
| *Ec*MaeB  R482A/Y510A | Forward | 5’ ATGGCGATTCAGAAACTGGGCTTGC 3’ |
|  | Reverse | 5’ TTCGATCACGTTCGGACGACCGATA 3’ |
| *Ec*MaeB  R475A/R482A/Y510A | Forward | 5’ GAGGCGTGGACCGAATACTTCCAGA 3’ |
|  | Reverse | 5’ TTTAAAGCGCGGATCGGATTCGTTA 3’ |
| *Bb*MaeB-SSB-1 | Forward | 5’ GAAAACCTGTATTTTCAGGGCATGGAAACCAAAACCGAAACCAAAAC 3’ |
|  | Reverse | 5’ AATCAGATAATCGCGGCCGAATTTAA 3’ |
| *Bb*MaeB-SSB-2 | Forward | 5’ CGCGATTATCTGATTCCGAAACCGTTCGATACCCG 3’ |
|  | Reverse | 5’ CGCGATTATCTGATTCCGAAACCGTTCGATACCCG 3’ |

**Table S4 |** **X-ray crystallography data collection, processing, and refinement parameters.**

|  | ***Ec*MaeB_apo_** |
| --- | --- |
| **Data collection** |  |
| Beamline | BL44XU |
| Wavelength (Å) | 0.900 |
| Space group | *C*2 |
| *a, b, c* (Å) | 347.71, 200.29, 201.82 |
| *α, β, γ* (°) | 90.00, 101.35, 90.00 |
| Resolution (Å) | 49.47–3.85 (3.92–3.85) |
| *R*_merge_ (all I+ and I−) | 0.140 (1.315) |
| *R*_p.i.m_ (all I+ and I−) | 0.087 (0.798) |
| Completeness (%) | 99.1 (99.8) |
| Total reflections | 446,934 (23,142) |
| Unique reflections | 127,049 (6,295) |
| <*I/σ (I)*> | 9.1 (1.4) |
| CC_1/2_ | 0.994 (0.537) |
| **Refinement** |  |
| Resolution (Å) | 49.47–3.85 (3.92–3.85) |
| *R*_work_ (%)/*R*_free_ (%) | 19.54/24.00 |
| RMSD bond length (Å) | 0.0023 |
| RMSD bond angle (°) | 0.494 |
| Average *B*-factor (Å^2^) |  |
| Overall | 154.58 |
| Protein | 154.58 |
| Metal | 189.05 |
| Water | - |
| Ramachandran plot (%) |  |
| Favored | 96.46 |
| Allowed | 3.52 |
| Outliers | 0.01 |
| PDB code ID | 9KRW |

**Table S5 |** **Cryo-EM data collection parameters and model statistics of *Ec*MaeB.**

|  | ***Ec*MaeB_holo_** | ***Ec*MaeB_holo-ME_** | ***Ec*MaeB_acetyl-CoA_** | ***Ec*MaeB_acetyl-CoA-ME_** |
| --- | --- | --- | --- | --- |
| **Data collection and processing** |  |  |  |  |
| Magnification | 60,000 | 60,000 | 60,000 | 60,000 |
| Voltage (kV) | 200 | 200 | 300 | 300 |
| Electron exposure (e^−^/Å^2^) | 40.00 | 40.00 | 40.00 | 40.00 |
| Defocus range (µm) | −0.7 to −2.2 | −0.7 to −2.2 | −0.7 to −2.0 | −0.7 to −2.0 |
| Pixel size (Å) | 0.83 | 0.83 | 0.87 | 0.87 |
| Symmetry imposed | *D*3 | *C*1 | *D*3 | *C*1 |
| Initial particle images (no.) | 3,242,518 | 3,242,518 | 5,848,385 | 5,848,385 |
| Final particle images (no.) | 125,475 | 376,425 | 501,182 | 501,182 |
| Map resolution (Å) | 2.73 | 3.19 | 2.03 | 2.39 |
| FSC threshold | 0.143 | 0.143 | 0.143 | 0.143 |
| Map resolution range (Å) | 2.40–9.34 | 2.79–11.44 | 1.95–27.86 | 2.10–31.82 |
| **Refinement** |  |  |  |  |
| Initial model used (PDB code ID) | 9KRW | 9KRW | 9KRT | 9KRT |
| Model resolution (Å) | 2.90 | 3.53 | 2.11 | 2.47 |
| FSC threshold | 0.5 | 0.5 | 0.5 | 0.5 |
| Map sharpening *B*-factor (Å^2^) | 93.9 | 98.2 | 58.8 | 63.6 |
| Model composition |  |  |  |  |
| Non-hydrogen atoms | 5,822 | 6,760 | 5,874 | 6,758 |
| Protein residues | 758 | 878 | 758 | 878 |
| Water | 0 | 0 | 1 | 0 |
| Ligands | 2 | 4 | 3 | 4 |
| *B*-factors (Å^2^) |  |  |  |  |
| Protein | 92.61 | 68.33 | 50.94 | 33.96 |
| Ligand | 195.09 | 129.47 | 89.09 | 47.44 |
| R.m.s. deviations |  |  |  |  |
| Bond lengths (Å) | 0.004 (0) | 0.004 (0) | 0.002 (0) | 0.005 (0) |
| Bond angles (°) | 0.746 (1) | 0.735 (7) | 0.563 (4) | 0.776 (1) |
| Validation |  |  |  |  |
| MolProbity score | 2.45 | 2.30 | 2.11 | 2.21 |
| Clashscore | 16.37 | 12.48 | 7.53 | 5.36 |
| Ramachandran Favored (%) | 91.27 | 90.96 | 93.39 | 92.22 |
| Ramachandran Allowed (%) | 8.47 | 8.70 | 6.48 | 6.64 |
| Ramachandran Outliers (%) | 0.26 | 0.34 | 0.13 | 1.14 |

**Table S6 |** **Cryo-EM data collection parameters and model statistics of *Bb*MaeB.**

|  | ***Bb*MaeB_holo_** | ***Bb*MaeB_acetyl-CoA_** |
| --- | --- | --- |
| **Data collection and processing** |  |  |
| Magnification | 60,000 | 60,000 |
| Voltage (kV) | 200 | 200 |
| Electron exposure (e^−^/Å^2^) | 40.00 | 40.00 |
| Defocus range (µm) | −0.7 to −2.2 | −0.7 to −2.2 |
| Pixel size (Å) | 0.83 | 0.83 |
| Symmetry imposed | *D*3 | *C*1 |
| Initial particle images (no.) | 5,033,529 | 6,186,794 |
| Final particle images (no.) | 449,060 | 165,921 |
| Map resolution (Å) | 2.59 | 3.18 |
| FSC threshold | 0.143 | 0.143 |
| Map resolution range (Å) | 2.19–9.48 | 2.83–50.50 |
| **Refinement** |  |  |
| Initial model used (PDB code ID) | AlphaFold | 6ZNG |
| Model resolution (Å) | 2.86 | 3.25 |
| FSC threshold | 0.5 | 0.5 |
| Map sharpening *B*-factor (Å^2^) | 104.3 | 87.5 |
| Model composition |  |  |
| Non-hydrogen atoms | 5,856 | 22,847 |
| Protein residues | 761 | 2,888 |
| Water | 0 | 6 |
| Ligands | 1 | 14 |
| *B*-factors (Å^2^) |  |  |
| Protein | 44.83 | 50.25 |
| Ligand | 167.13 | 68.05 |
| R.m.s. deviations |  |  |
| Bond lengths (Å) | 0.002 (0) | 0.003 (0) |
| Bond angles (°) | 0.549 (1) | 0.508 (0) |
| Validation |  |  |
| MolProbity score | 1.67 | 2.23 |
| Clashscore | 7.56 | 10.49 |
| Ramachandran plot (%) |  |  |
| Favored | 96.18 | 95.65 |
| Allowed | 3.82 | 4.18 |
| Outliers | 0.00 | 0.17 |

**
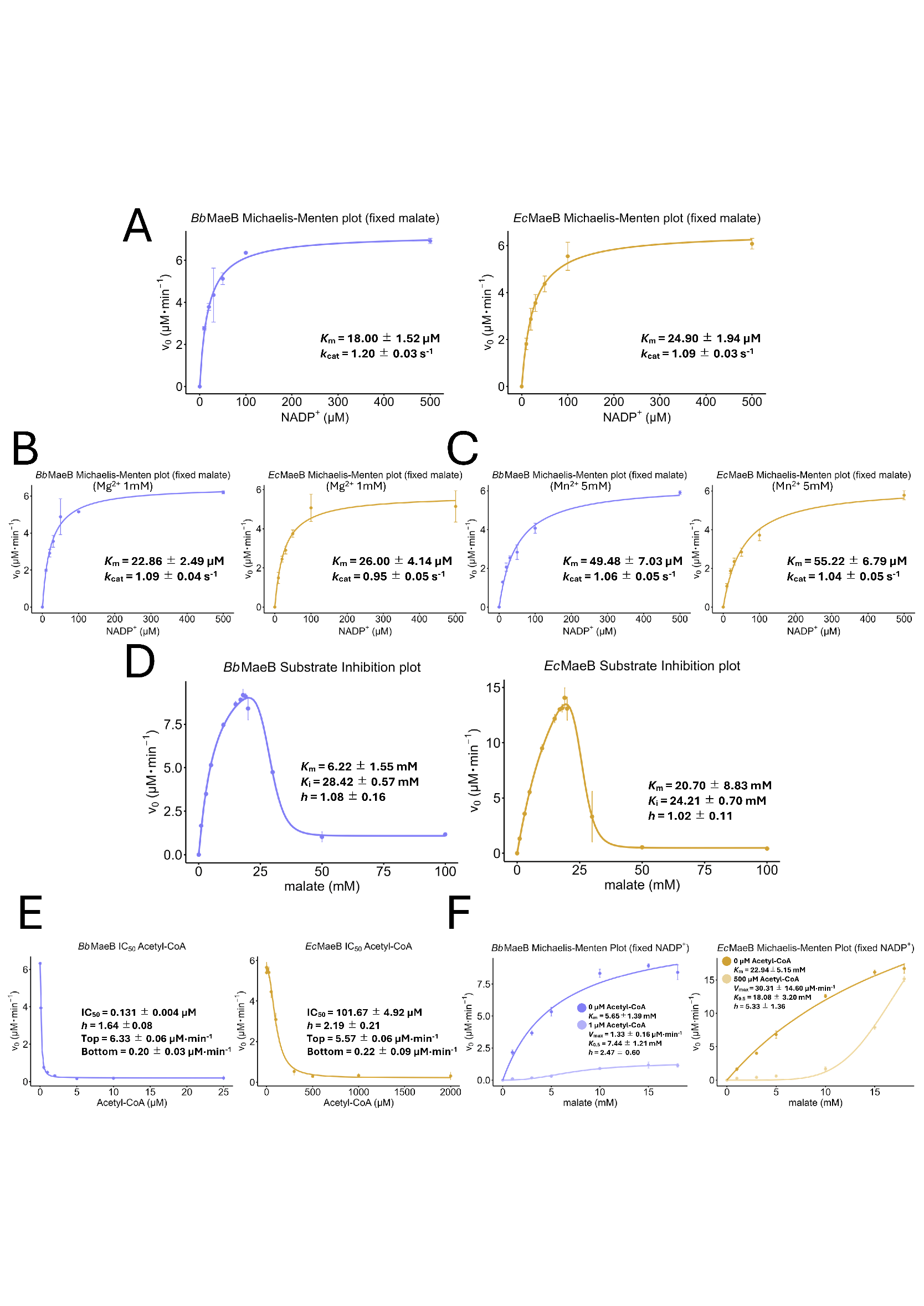
Figures**

**Figure S1 |** **Enzymatic assays for *Ec*MaeB and *Bb*MaeB. A)** Michaelis-Menten plots for MaeBs when the concentration of malate was fixed to be 5 mM. **B)** Michaelis-Menten plots for MaeBs when 1 mM MgCl_2_ was used and the concentration of malate was fixed to be 0.5 mM. **C)** Michaelis-Menten plots for MaeBs when 5 mM MnCl_2_ was used and the concentration of malate was fixed to be 0.5 mM. **D)** Substrate inhibition plots for MaeBs (NADP^+^ was fixed to be 0.5 mM). **E)** Acetyl-CoA inhibition plots for MaeBs. **F)** Michaelis-Menten and empirical Hill plots for MaeBs (under the condition of 0.5 mM NADP^+^). For the data obtained without acetyl-CoA, Michaelis–Menten fitting was applied instead of the substrate inhibition model used in panel C. As a result, the estimated *K*_m_ values differ slightly between the two analyses, but the differences fall within the margin of error.


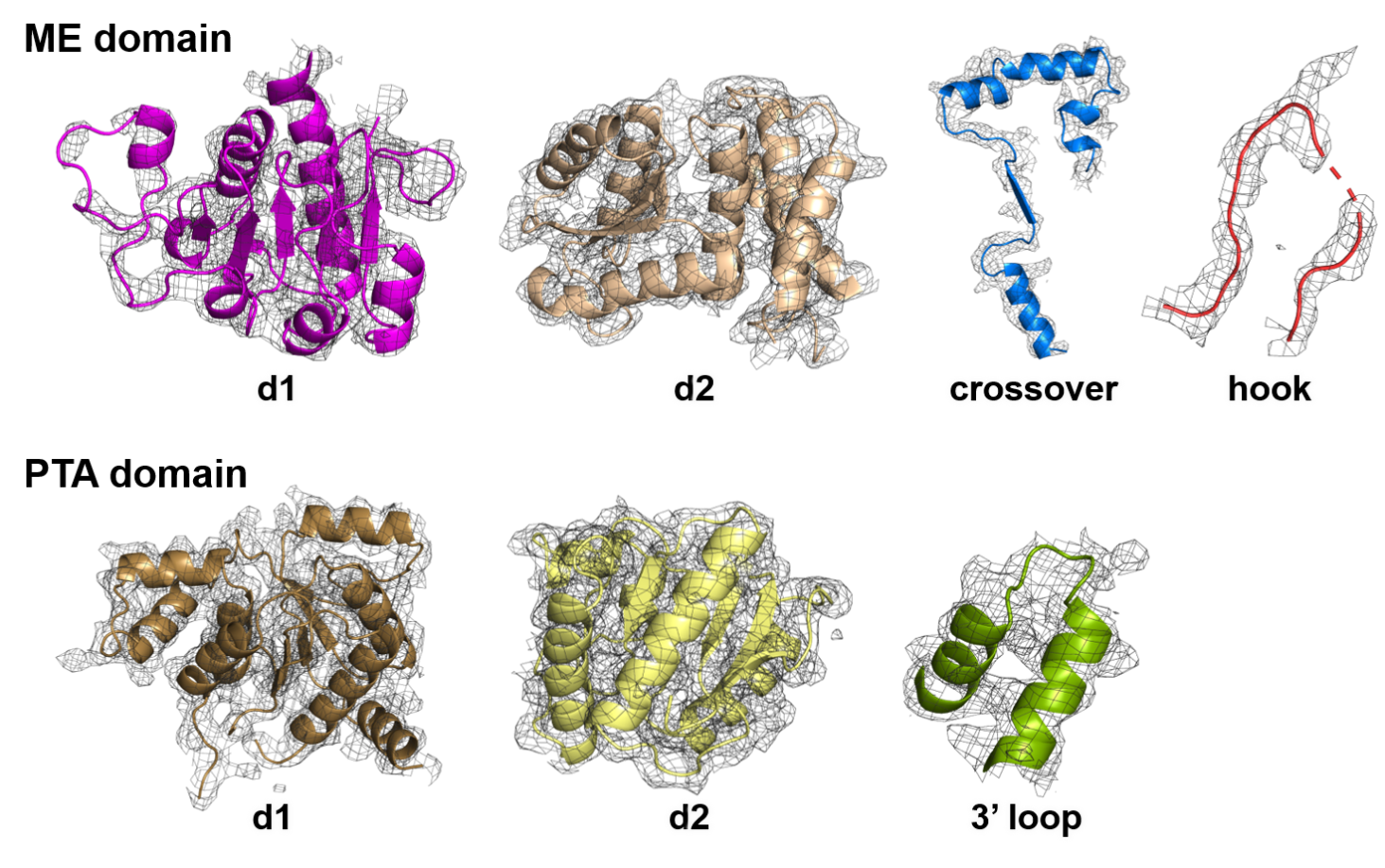


**Figure S2 |** **Representative electron density maps of the *Ec*MaeB crystal structure.** The 2m*F*_o_-D*F*_c_ maps around each domain are shown as a gray mesh contoured at 1.0σ.


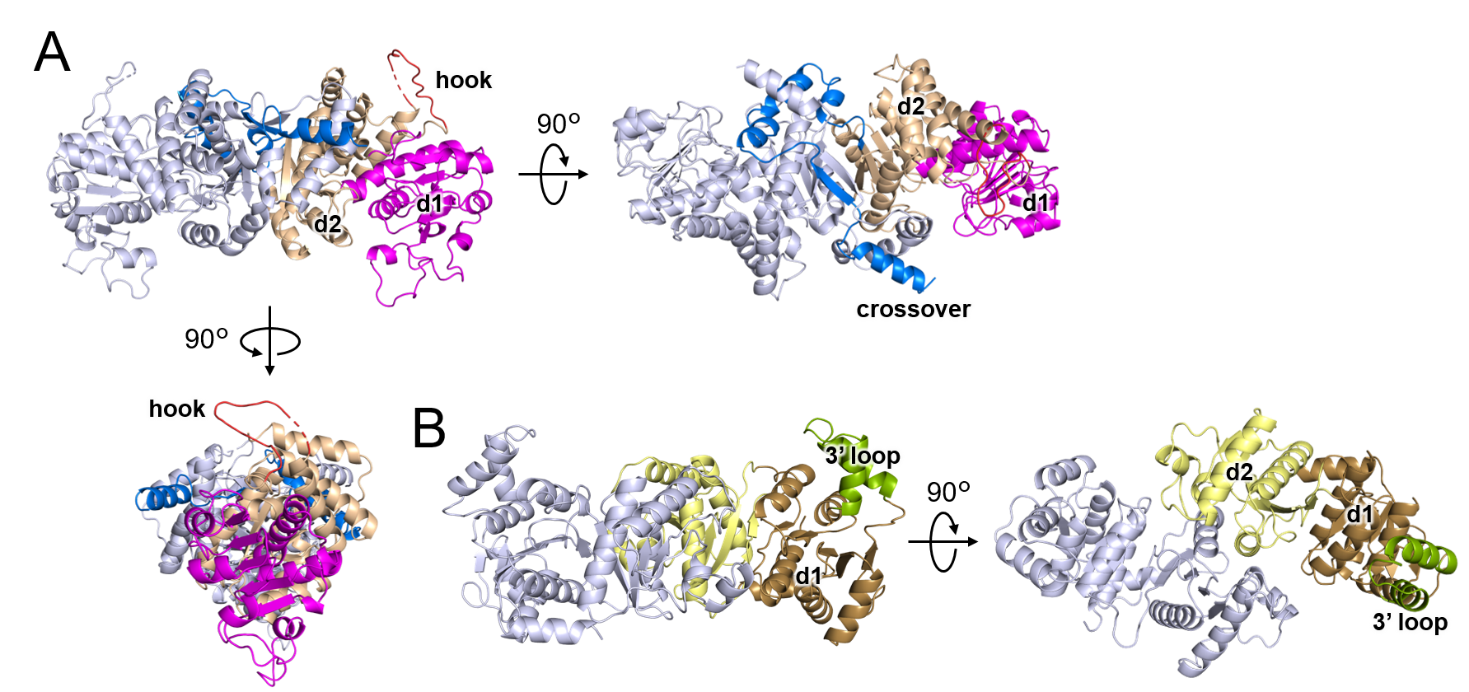


**Figure S3 |** **Detailed description of the *Ec*MaeB crystal structure. A)** Structure of the dimer of the ME domain. Crossover (blue), d1 (magenta), d2 (wheat) and hook (red) subdomains are highlighted in a different color. **B)** Structure of the dimer of the PTA domain. Subdomains d1 (sand), d2 (yellow), and 3’ loop (green) are highlighted in a different color.


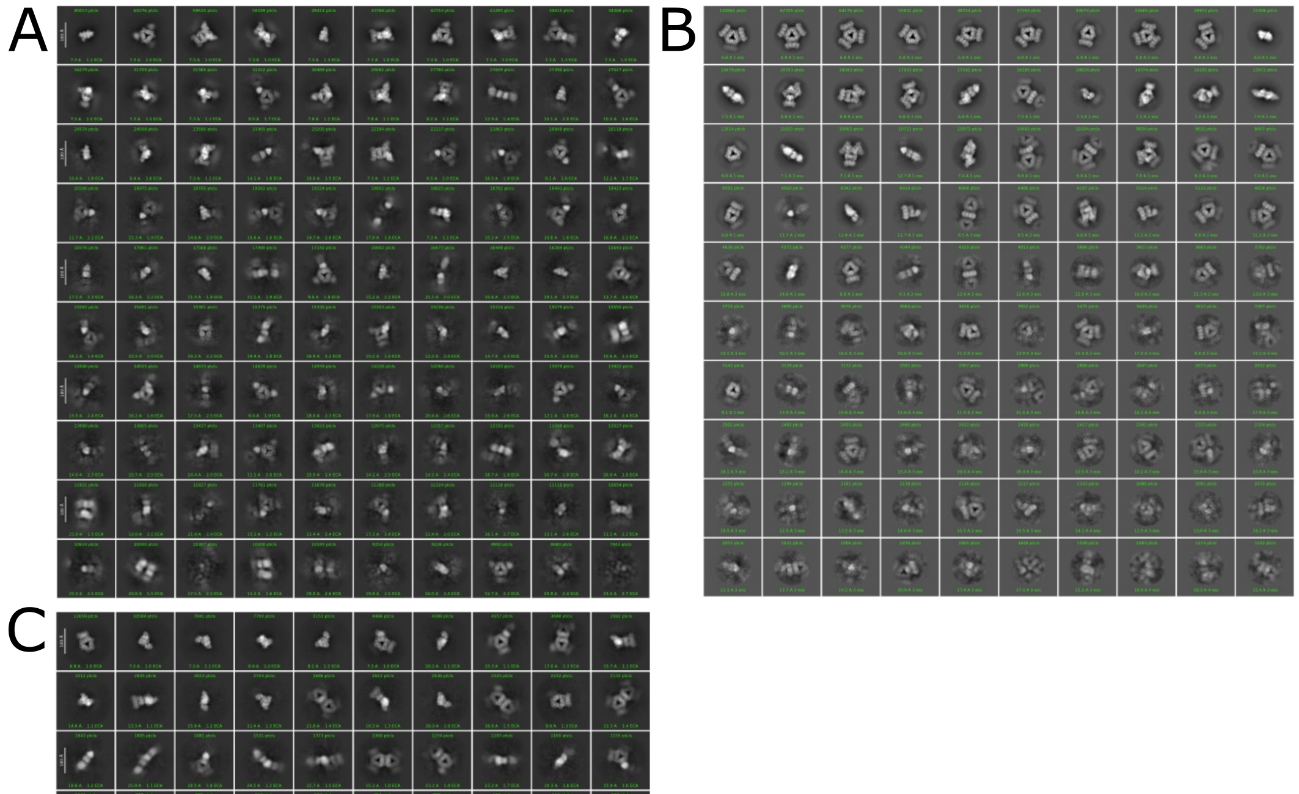


**Figure S4 |** **Representative particles after 2D classification. A)** *Ec*MaeB_holo_. **B)** *Bb*MaeB_holo_. **C)** *Bb*MaeB_acetyl-CoA_


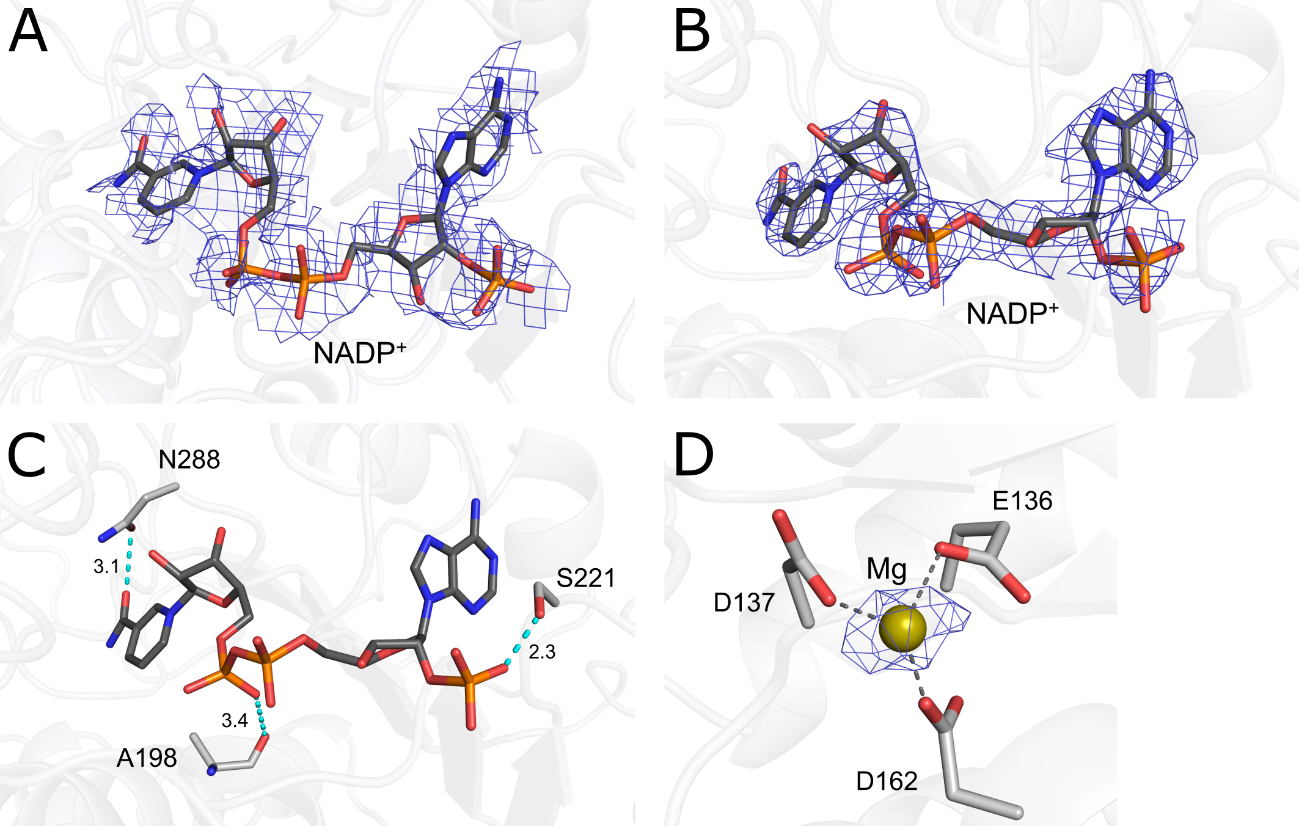


**Figure S5 |** **The cryo-EM maps of cofactors. A)** The EM density of NADP^+^ in the *Ec*MaeB holo form ME domain dimer (*Ec*MaeB_holo-ME_). The EM density of NADP^+^ is shown as a mesh, and a NADP^+^ molecule that fit in the density is shown as a stick model colored by elements. **B)** The EM density of NADP^+^ in *Ec*MaeB acetyl-CoA bound form ME domain dimer (*Ec*MaeB_acetyl-CoA-ME_). The EM density of NADP^+^ is shown as a mesh, and a NADP^+^ molecule that fits in the density is shown as a stick model colored by elements. **C)** Schematic diagram for the interaction between NADP^+^ and *Ec*MaeB_acetyl-CoA-ME_. The numbers next to dot-dash lines indicate the distance between atoms forming an interaction (Å). **D)** The EM density of Mg ion in *Ec*MaeB_acetyl-CoA-ME_. Coordination bonds are shown by gray dot-dash lines.


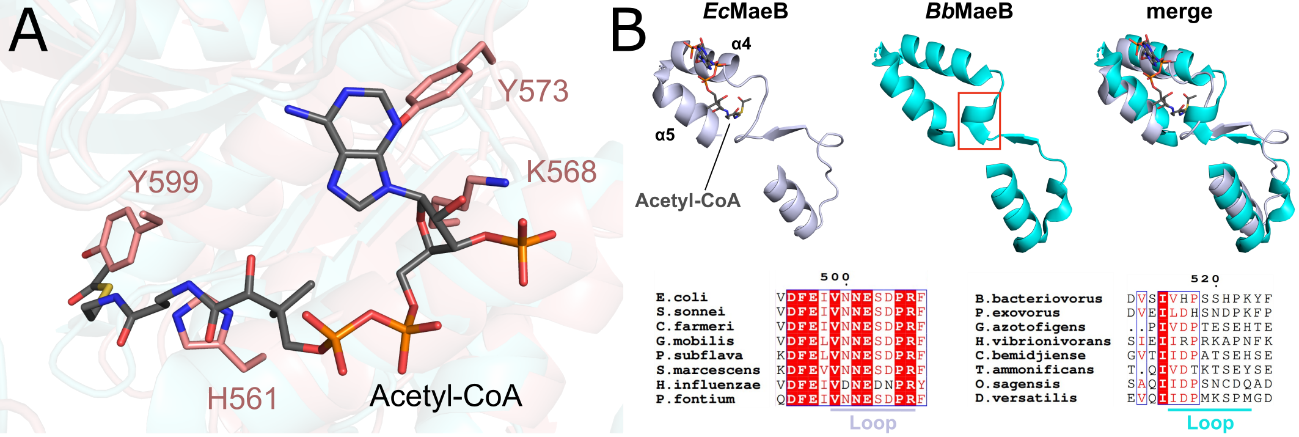


**Figure S6 |** **Comparison of acetyl-CoA binding sites between *Ec*MaeB and *Bb*MaeB. A)** Structural overlay of *Ec*MaeB_apo_ (salmon) and *Bb*MaeB acetyl-CoA bound form (PDB code ID: 6ZNG) (cyan). At the acetyl-CoA binding site in *Bb*MaeB, *Ec*MaeB has bulky amino acids, such as H561, K568, Y573, and Y599. **B)** Structural comparison of near the acetyl-CoA binding site of *Ec*MaeB (upper). An acetyl-CoA inserts its thioacetyl group to the loop (V499–R506) in *Ec*MaeB_acetyl-CoA_ (left), while part of the loop (H517–H521) forms a 3_10_-helix in *Bb*MaeB acetyl-CoA bound form (PDB code ID: 6ZNG) (center). Superposition of *Ec*MaeB and *Bb*MaeB shows that partly secondary structure interferes the binding of acetyl-CoA to this site in *Bb*MaeB. Sequence alignments of hybrid-type MEs (lower).


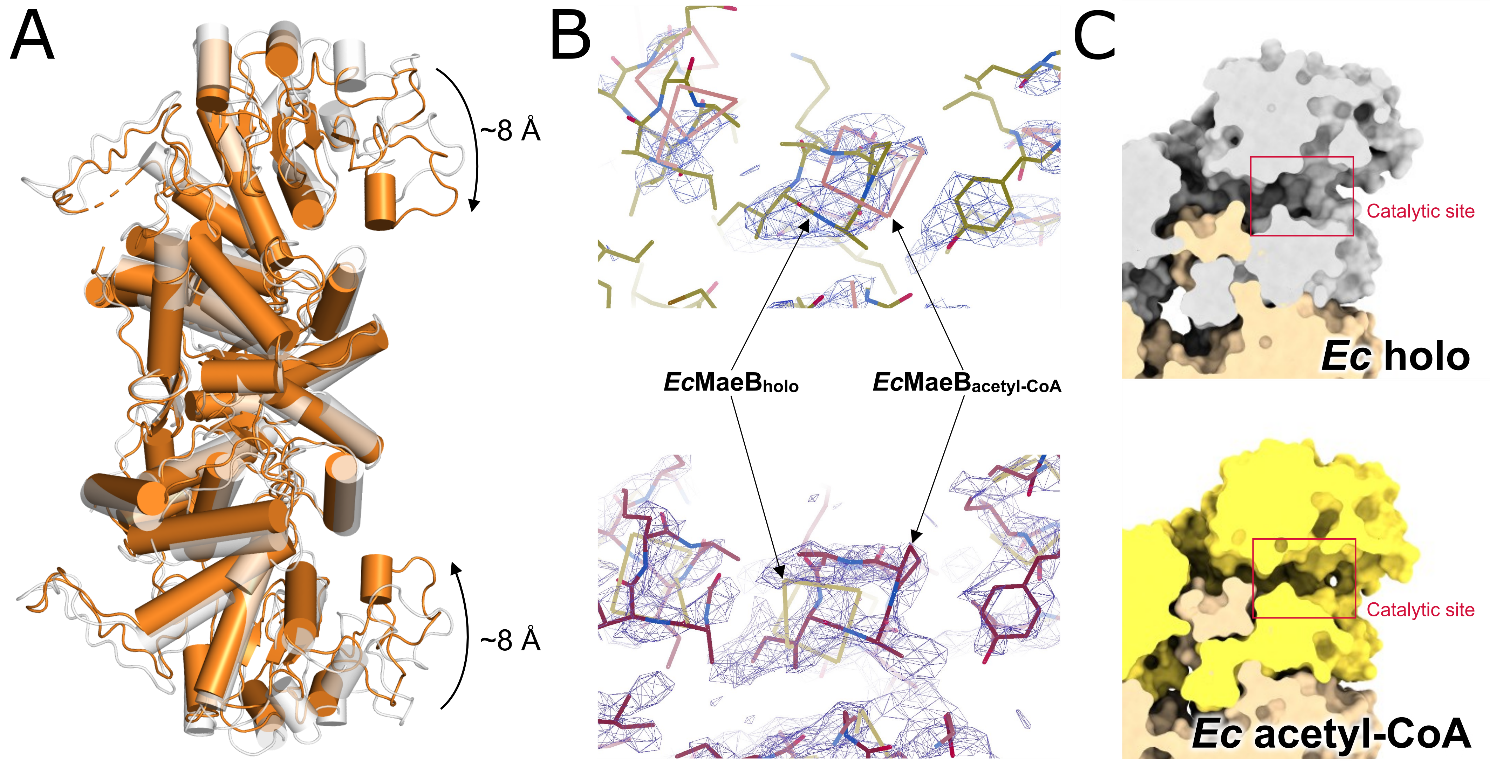


**Figure S7 | Structural comparison of the ME domain dimers *Ec*MaeB. A)** *Ec*MaeB_holo_ (white) and *Ec*MaeB_acetyl-CoA_ (orange). For structural comparison, the ME domain dimer in two protomers are superimposed (Cα RMSD = 2.31 Å over 787 atoms). The numbers next to the arrows indicate the distance the ME domains moved (Å). **B)** The superposition of the ME domain dimers between *Ec*MaeB_holo_ and *Ec*MaeB_acetyl-CoA_. The visualized cryo-EM maps are the cryo-EM map of *Ec*MaeB_holo_ (upper) and that of *Ec*MaeB_acetyl-CoA_ (lower). **C)** Comparison of the catalytic sites of *Ec*MaeB_holo_ (upper) and that of *Ec*MaeB_acetyl-CoA_ (lower). In *Ec*MaeB_acetyl-CoA_, the space near the catalytic site is narrower due to the binding of acetyl-CoA.


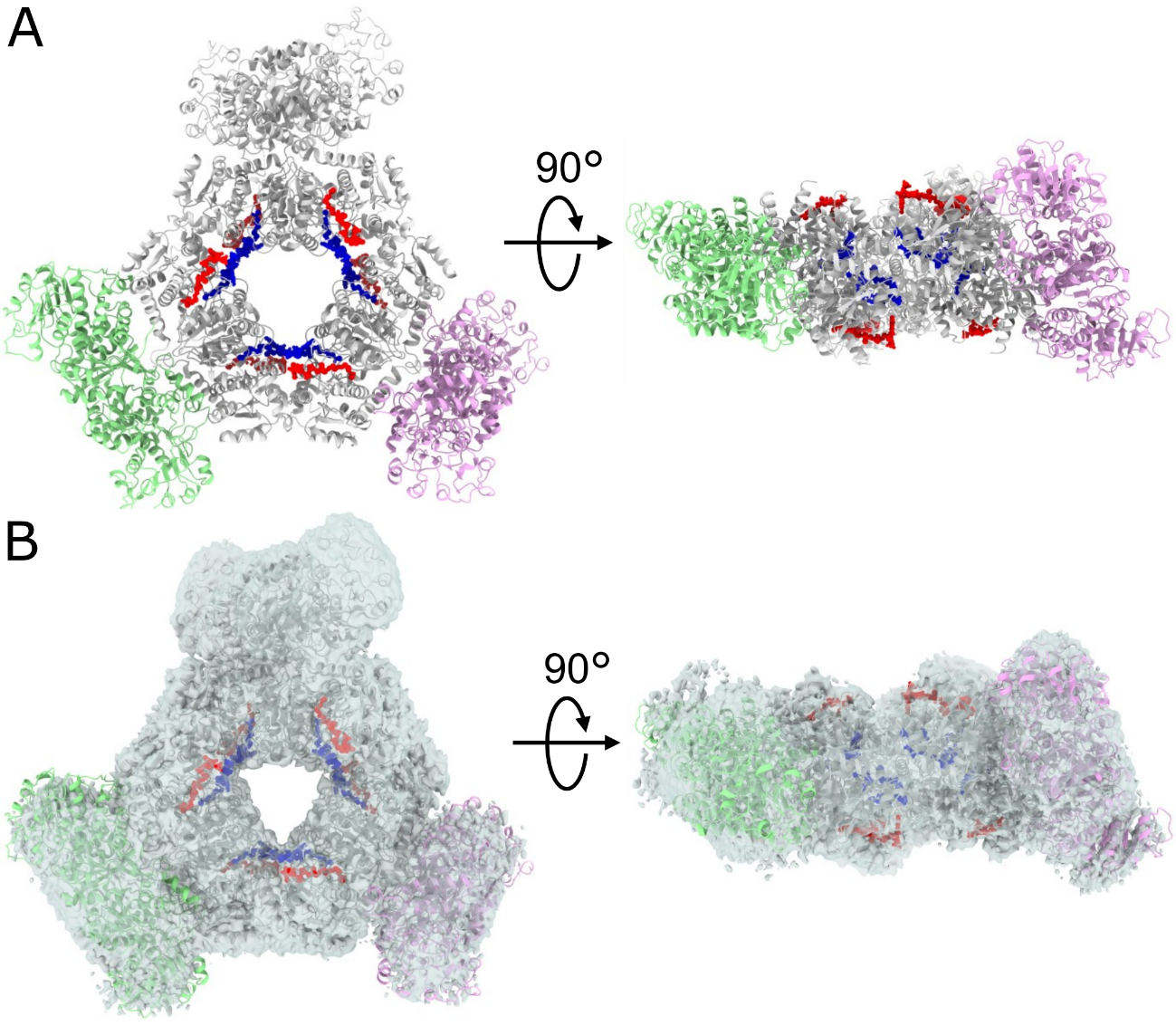


**Figure S8 |** **Difference of the conformation of the ME domain dimers in *Bb*MaeB_acetyl-CoA_. A)** Structural model consisting of three parts: PTA domain and one ME domain dimer (gray), one ME domain dimer (green), and the other ME domain dimer (pink). **B)** Superposition of structural model and cryo-EM density map.


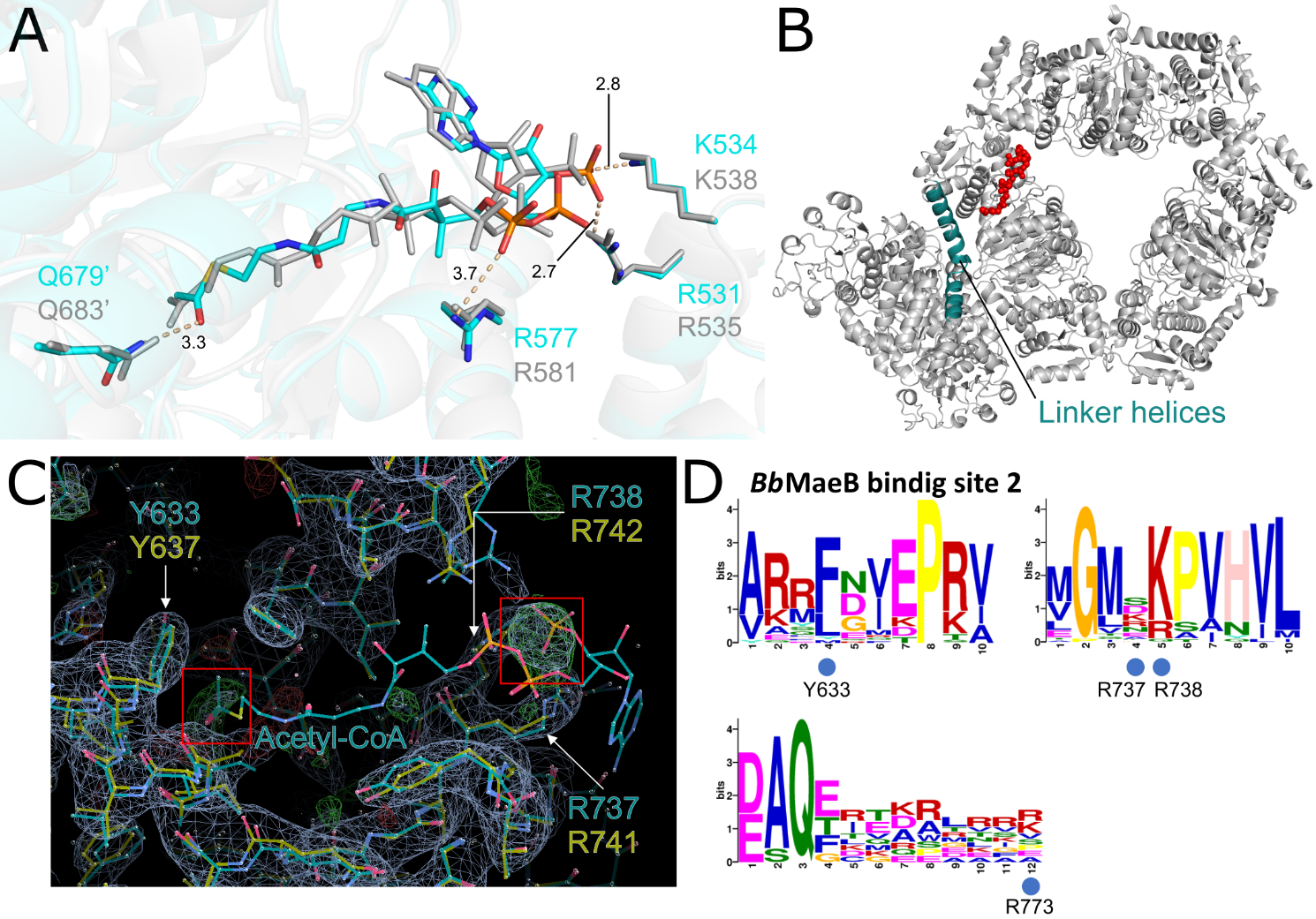


**Figure S9 | Acetyl-CoA binding sites of *Bb*MaeB. A)** Comparison of acetyl-CoA binding sites in *Bb*MaeB-HD (gray) and binding site 1 in *Bb*MaeB_acetyl-CoA_ (cyan) and schematic diagram for the interaction between acetyl-CoA and amino acids. Acetyl-CoA molecules are shown as a stick model, and acetyl-CoA in *Bb*MaeB_acetyl-CoA_ is colored by elements. The numbers next to dot-dash lines indicate the distance between atoms forming an interaction (Å). **B)** The structural model of acetyl-CoA at the binding site 2 in *Bb*MaeB_acetyl-CoA_ and linker helices. Acetyl-CoA at the binding site 2 is colored red and the linker helices are colored teal, respectively. **C)** Superposition of *Bb*MaeB_acetyl-CoA_ (cyan) and *Bb*MaeB-HD acetyl-CoA bound form (PDB code ID: 6ZNG) (yellow) near the binding site 2. The 2*mF*_o_–*DF*_c_ (1.0σ) map of *Bb*MaeB-HD acetyl-CoA bound form is visualized. The electron density in the area enclosed by the red square may be derived from acetyl-CoA. **D)** The acetyl-CoA binding motif of the binding site 2 in *Bb*MaeB.


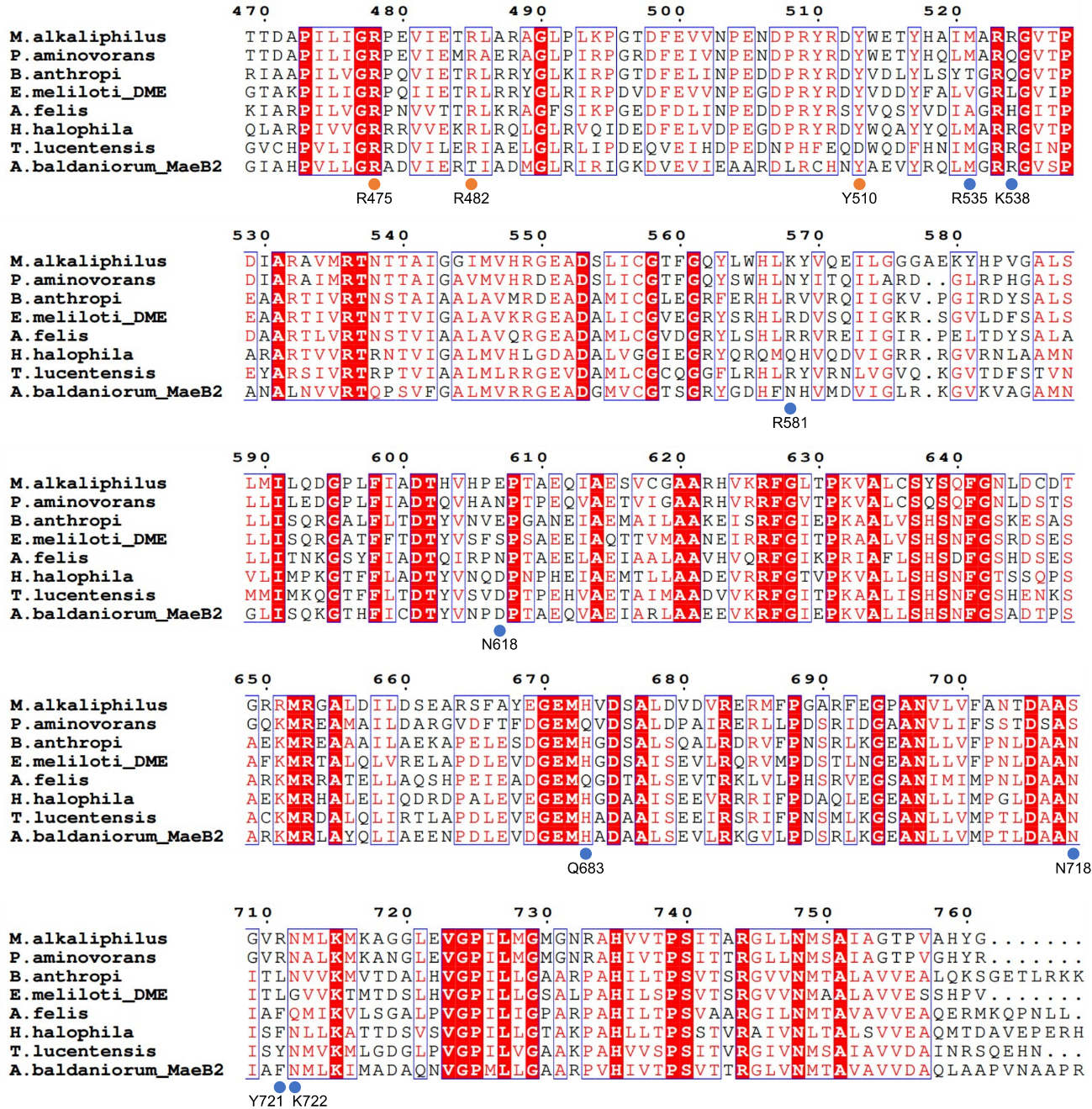


**Figure S10 | Sequence alignment of hybrid-type MEs from an unclassified clade near clade 1.** Orange circles: Residues which are involved in binding of acetyl-CoA in *Ec*MaeB. Blue circles: Residues which are involved in binding of acetyl-CoA in *Bb*MaeB.


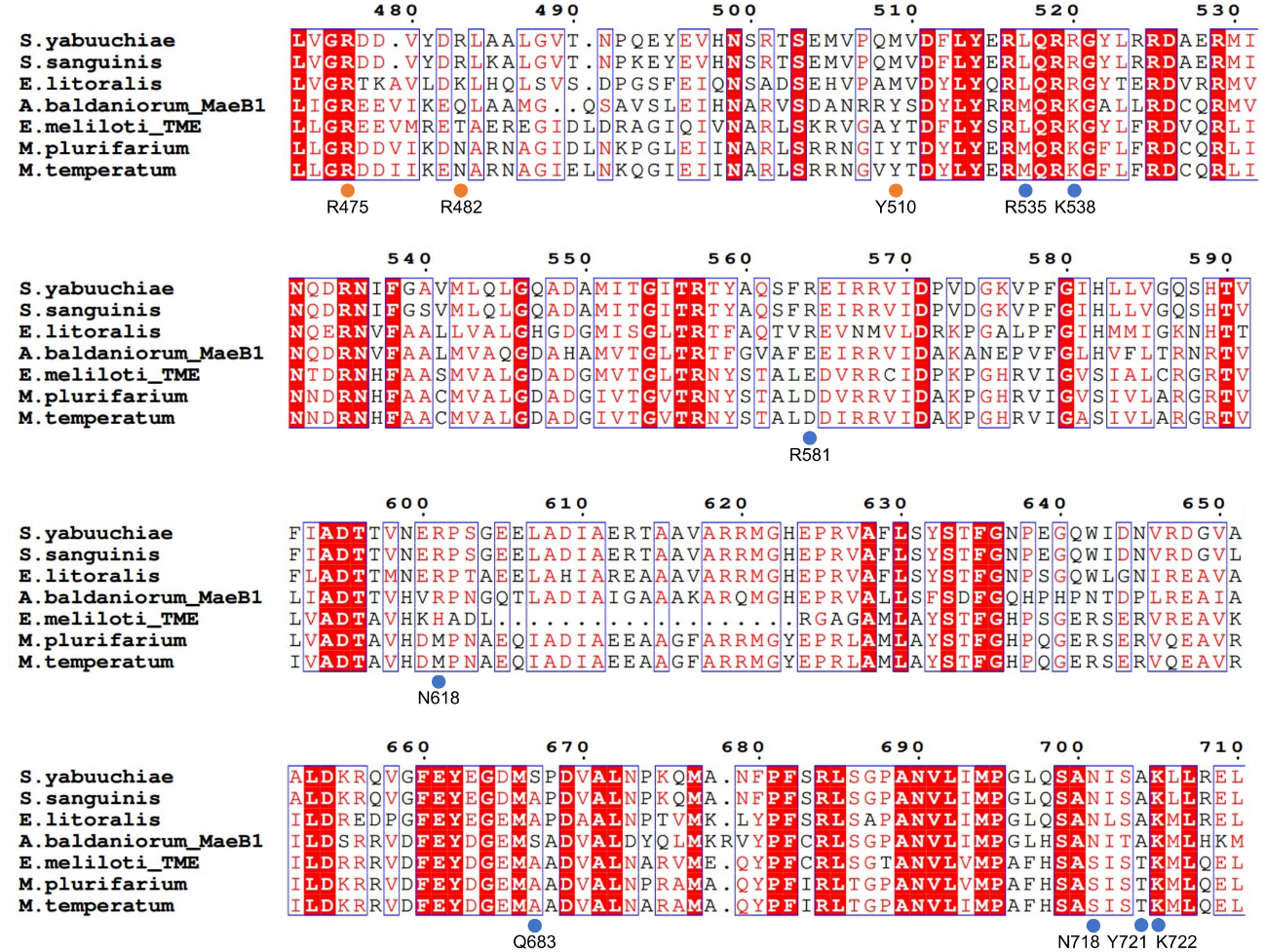


**Figure S11 | Sequence alignment of hybrid-type MEs from an unclassified clade near clade 2.** Orange circles: Residues which are involved in binding of acetyl-CoA in *Ec*MaeB. Blue circles: Residues which are involved in binding of acetyl-CoA in *Bb*MaeB.


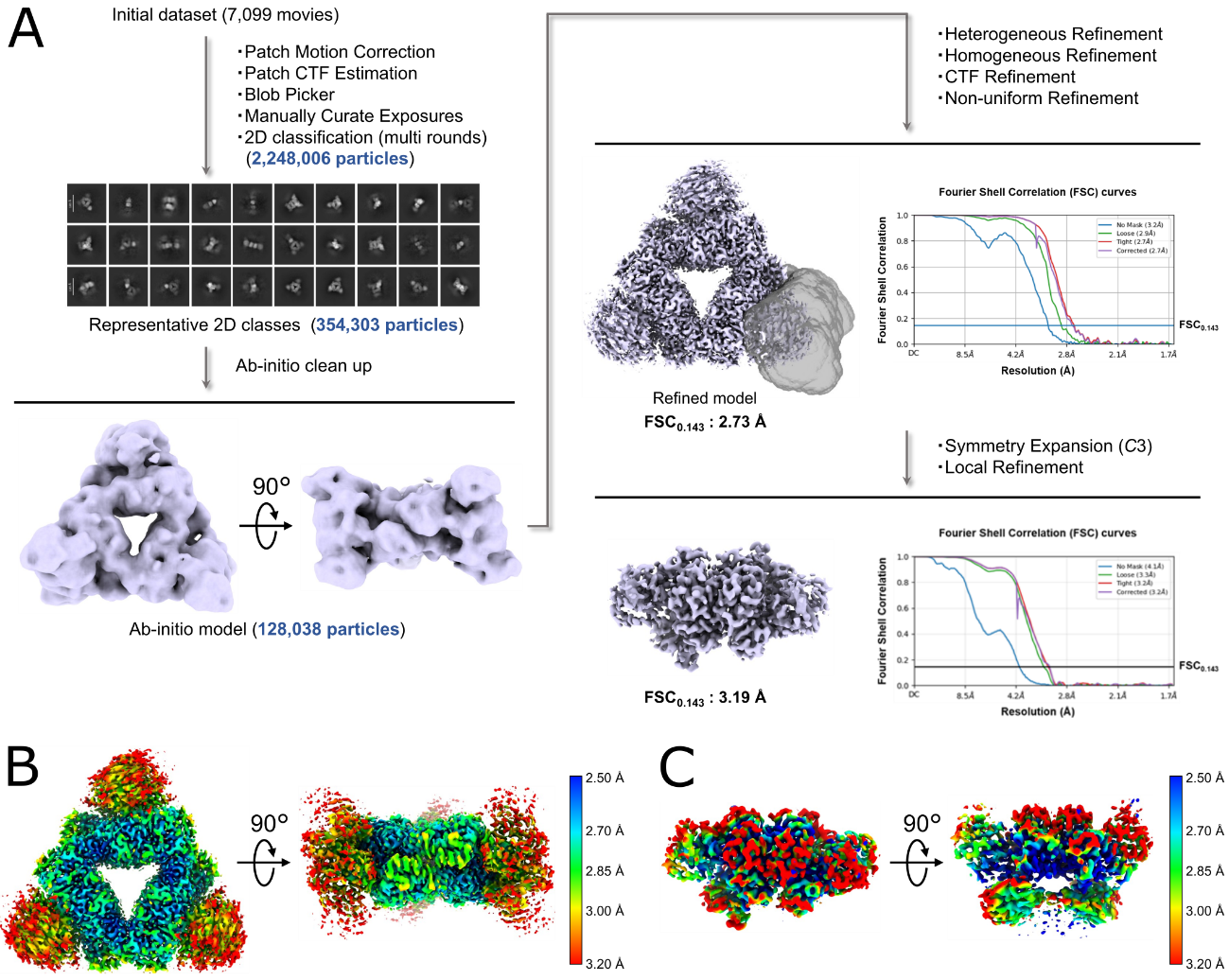


**Figure S12 | Cryo-EM analysis of *Ec*MaeB_holo_ and *Ec*MaeB_holo-ME_.** **A)** An overview of the workflow for the cryo-EM data processing. 7,099 movies were collected and processed for the cryo-EM reconstruction. Various stages of data processing include 2D classification and *ab*-*initio* clean up using CryoSPARC. The representative 2D classes are shown and the major 3D class is used for refinement. The map of *Ec*MaeB_holo_ calculated after Non-uniform Refinement has a FSC_0.143_ of 2.73 Å. The map of *Ec*MaeB_holo-ME_ calculated after Local Refinement has a FSC_0.143_ of 3.19 Å. Number of particles used at key stages of data processing are shown in blue. **B)** Local resolution of *Ec*MaeB_holo_. **C)** Local resolution of *Ec*MaeB_holo-ME_.


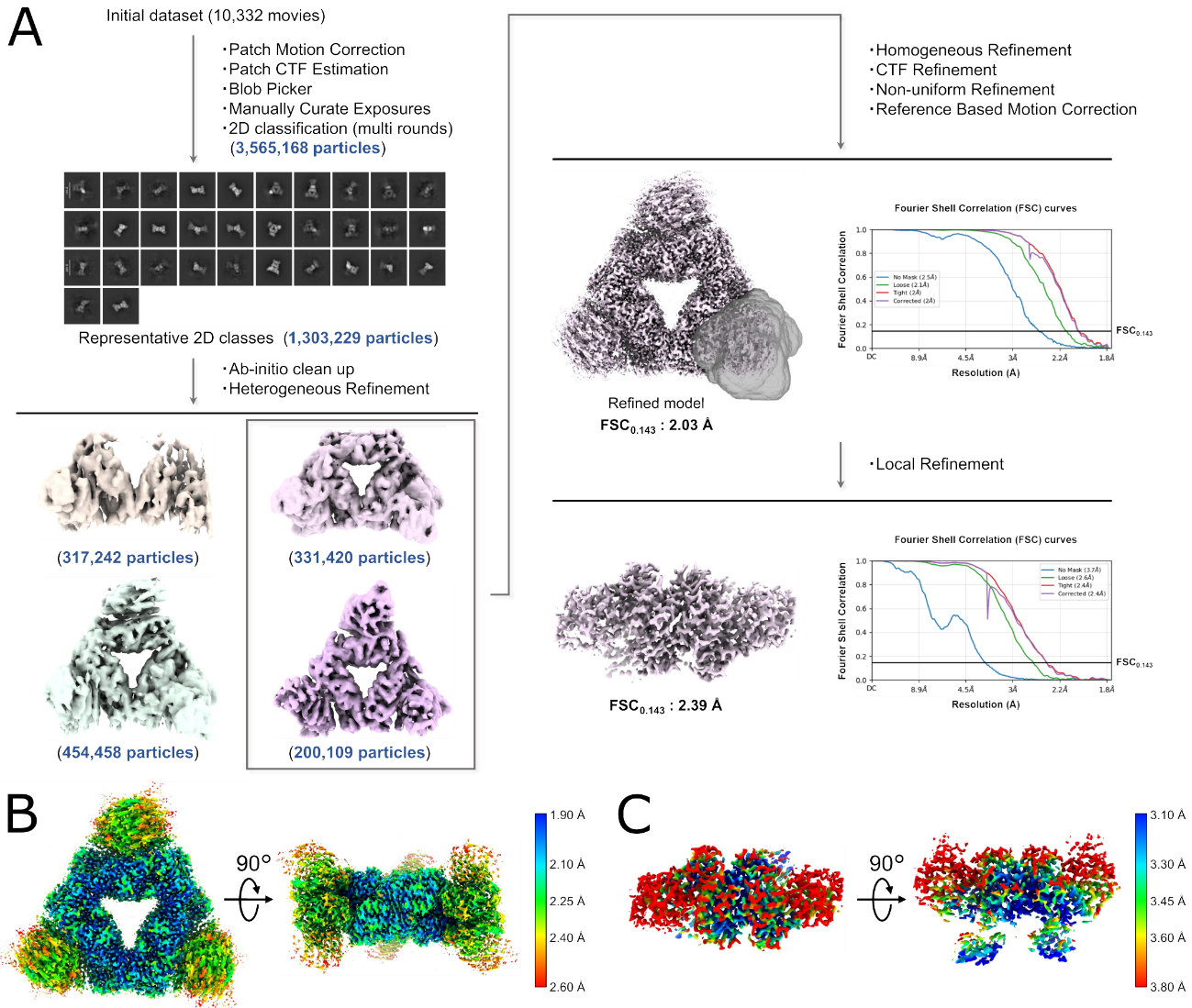


**Figure S13 | Cryo-EM analysis of *Ec*MaeB_acetyl-CoA_ and *Ec*MaeB_acetyl-CoA-ME_.** **A)** An overview of the workflow for the cryo-EM data processing. 10,332 movies were collected and processed for the cryo-EM reconstruction. Various stages of data processing include 2D classification using CryoSPARC. The representative 2D classes are shown and the major 3D class is used for refinement. The map of *Ec*MaeB_acetyl-CoA_ calculated after Non-uniform Refinement has a FSC_0.143_ of 2.03 Å. The map of *Ec*MaeB_acetyl-CoA-ME_ calculated after Local Refinement has a FSC_0.143_ of 2.39 Å. Number of particles used at key stages of data processing are shown in blue. **B)** Local resolution of *Ec*MaeB_acetyl-CoA_. **C)** Local resolution of *Ec*MaeB_acetyl-CoA-ME_.


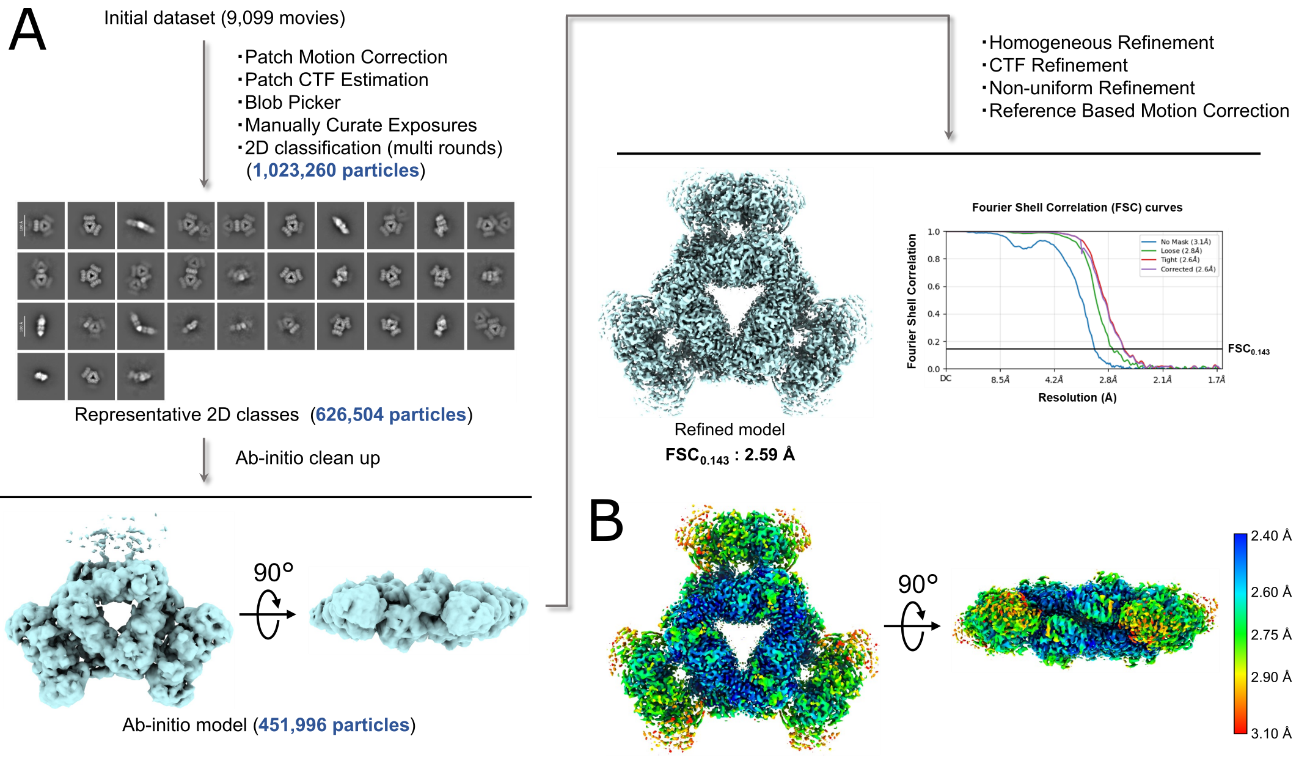


**Figure S14 | Cryo-EM analysis of *Bb*MaeB_holo_.** **A)** An overview of the workflow for the cryo-EM data processing. 9,099 movies were collected and processed for the cryo-EM reconstruction. Various stages of data processing include 2D classification and *ab*-*initio* clean up using CryoSPARC. The representative 2D classes are shown and the major 3D class is used for refinement. The map calculated after Non-uniform Refinement has a FSC_0.143_ of 2.59 Å. Number of particles used at key stages of data processing are shown in blue. **B)** Local resolution of the global map.

**
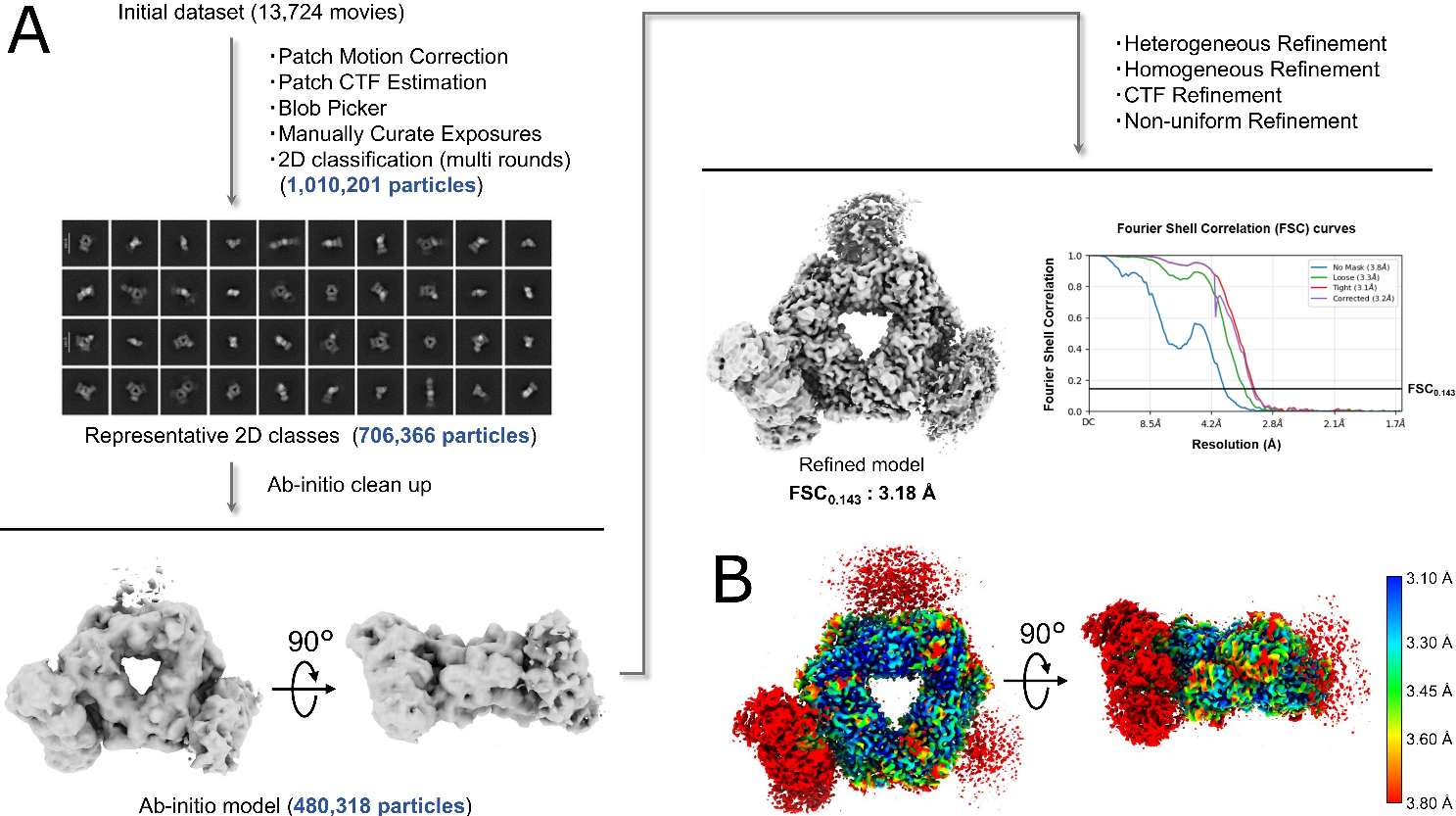
**

**Figure S15 | Cryo-EM analysis of *Bb*MaeB_acetyl-CoA_.** **A)** An overview of the workflow for the cryo-EM data processing. 13,724 movies were collected and processed for the cryo-EM reconstruction. Various stages of data processing include 2D classification and *ab*-*initio* clean up using CryoSPARC. The representative 2D classes are shown and the major 3D class is used for refinement. The map calculated after Non-uniform Refinement has a FSC_0.143_ of 3.18 Å. Number of particles used at key stages of data processing are shown in blue. **B)** Local resolution of the global map.
